# Extracellular vesicle mimetics as delivery vehicles for oligonucleotide-based therapeutics and plasmid DNA

**DOI:** 10.3389/fbioe.2024.1437817

**Published:** 2024-10-17

**Authors:** Anastasiya Oshchepkova, Ivan Chernikov, Svetlana Miroshnichenko, Olga Patutina, Oleg Markov, Innokenty Savin, Yaroslav Staroseletz, Mariya Meschaninova, Pavel Puchkov, Sergey Zhukov, Maxim Kupryushkin, Mikhail Maslov, Aleksandra Sen’kova, Valentin Vlassov, Elena Chernolovskaya, Marina Zenkova

**Affiliations:** ^1^ Laboratory of Nucleic Acids Biochemistry, Institute of Chemical Biology and Fundamental Medicine SB RAS, Novosibirsk, Russia; ^2^ Laboratory of RNA Chemistry, Institute of Chemical Biology and Fundamental Medicine SB RAS, Novosibirsk, Russia; ^3^ Lomonosov Institute of Fine Chemical Technologies, MIREA—Russian Technological University, Moscow, Russia; ^4^ Laboratory of Nucleic Acids Chemistry, Institute of Chemical Biology and Fundamental Medicine SB RAS, Novosibirsk, Russia

**Keywords:** extracellular vesicle mimetics, cytochalasin B, multidrug resistance, melanoma, siRNA, antisense oligonucleotide (ASO), immunostimulatory RNA (isRNA), plasmid DNA

## Abstract

**Introduction:**

Small membrane particles called extracellular vesicles (EVs) transport biologically active cargo between cells, providing intercellular communication. The clinical application of EVs is limited due to the lack of scalable and cost-effective approaches for their production and purification, as well as effective loading strategies.

**Methods:**

Here we used EV mimetics produced by cell treatment with the actin-destabilizing agent cytochalasin B as an alternative to EVs for the delivery of therapeutic nucleic acids.

**Results:**

Cytochalasin-B-inducible nanovesicles (CINVs) delivered a fully modified N-(methanesulfonyl)- or mesyl (µ-) antisense oligonucleotide to B16 melanoma cells, selectively decreasing the level of target microRNA-21 with effectiveness comparable to that observed upon Lipofectamine 2000-mediated delivery. The efficiency of the CINV-mediated delivery of plasmid DNA encoding EGFP varied depending on the type of recipient cells. Surprisingly, under experimental conditions, CINVs were unable to deliver both modified and natural short RNA duplexes—small interfering RNA and immunostimulatory RNA—probably due to their poor loading into CINVs.

**Discussion:**

CINVs demonstrated unique properties for the delivery of therapeutic nucleic acids, especially for antisense oligonucleotide-based therapy.

## 1 Introduction

An important part of modern biomedical research is focused on creating drug delivery systems (Liu et al., 2021; Gao et al., 2023; Golubovic et al., 2023) that can slow down the rate of drug elimination and degradation in the body as well as promote tissue-specific accumulation. Synthetic drug carriers are a diverse set of delivery systems whose main advantage is a controlled and well-standardized manufacturing process. However, they often have undesirable side effects, such as immunogenicity and toxicity (Sharma et al., 2021; Braatz et al., 2022; Sairam et al., 2023). Natural carriers (Lutz et al., 2019; Wang and Liu, 2021; Choi et al., 2023; Nguyen et al., 2023) appear to be more biocompatible; however, their preparation and purification are usually complicated.

Extracellular vesicles (EVs) are spherical membrane structures secreted by cells into the extracellular milieu during various biological processes (Jeppesen et al., 2023; Raposo and Stahl, 2023) and cannot replicate on their own (Welsh et al., 2024). The natural function of EVs is the transfer of biologically active cargo from one cell to another (Raposo and Stahl, 2024). Nowadays, EVs are receiving increased attention for the development of drug delivery systems (Liu et al., 2023; Lu et al., 2023; Oshchepkova et al., 2023; Wang L. et al., 2023; Whitley and Cai, 2023; Zeng et al., 2023). However, they still cannot be fully considered for clinical use because of difficulties with isolation and purification procedures (Clos-Sansalvador et al., 2022; Yakubovich et al., 2022; Havers et al., 2023; Hendrix et al., 2023). Moreover, the considerable heterogeneity of EVs and their contamination with non-vesicular nanoparticles and protein aggregates during isolation (Nieuwland et al., 2022; Jeppesen et al., 2023) make it challenging to standardize EV preparations. Another obstacle to using EVs for drug delivery is the long-term storage problems (Gelibter et al., 2022; Görgens et al., 2022).

The production of EV mimetics may be an advantageous alternative to naturally released EVs (Xu et al., 2023). EV mimetics are EV-like particles produced by artificial manipulations with natural membrane systems (Welsh et al., 2024), for example, by cell extrusion through porous filters of varying sizes (Ilahibaks et al., 2019; Lee et al., 2020). The composition of extruded cell vesicles is more similar to that of parent cells than that of natural EVs (Sayyed et al., 2023; Wang X. et al., 2023). The extrusion of specific organelles, such as endosomes (Guo et al., 2021), rather than whole cells, results in the formation of vesicles that more closely resemble EVs. Cell extrusion provides an efficient approach for the production of vesicles; however, severe mechanical stress may damage the structure of vesicle proteins, reducing their potential for targeted drug delivery.

Chemical stimulation can be used to boost the release of EVs (Debbi et al., 2022; Erwin et al., 2023). Boosting is based on the stimulation of certain pathways of EV biogenesis. Thus, the enhanced release of EVs, presumably originating from the endocytic compartments, was achieved after simultaneous inhibition of glycolysis and oxidative phosphorylation by cell treatment with sodium iodoacetate along with 2,4-dinitrophenol (Ludwig et al., 2020). Bafilomycin A1 enhances the release of EVs originating from the late endosomal compartments, whereas homosalate is supposed to activate the secretion of EVs originating from the plasma membrane (Grisard et al., 2022).

Chemically induced EV mimetics are formed by mechanisms unrelated to the natural pathways of EV biogenesis. For example, some chemical agents (paraformaldehyde, N-ethylmaleimide, *etc.*) induce irreversible plasma membrane blebbing (Thone and Kwon, 2020). Vesicularization can also be induced by cell treatment with actin-destabilizing agents such as cytochalasins or latrunculins. These vesicles or blebs can be classified as EV mimetics, since it is unlikely that the secretion of natural EVs can be enhanced in cells in a fixed physiological state or in the absence of a normal actin network.

This article reports the use of EV mimetics obtained by cell treatment with cytochalasin B (Cyt B), which have been named cytochalasin-B-inducible nanovesicles (CINVs) (Oshchepkova et al., 2019; Oshchepkova et al., 2021), for the delivery of nucleic acids into eukaryotic cells. It is worth mentioning that CINVs differ from cell membrane-coated nanoparticles (Wang and Liu, 2021) in that they partially retain the internal contents of their parent cells. In this context, CINVs are more reminiscent of cells or EVs that have their own biologically active internal content, which is absent in cell plasma membrane-based delivery systems. We examined whether CINVs could implement the functional delivery of different types of therapeutic nucleic acids (tNAs) into cells: N-(methanesulfonyl)- (mesyl- or µ-) antisense oligonucleotide targeted to microRNA-21 (µ-ON-21); small interfering RNA (siRNA) targeted to multidrug resistance gene 1 (*MDR1*); immunostimulatory RNA (isRNA); and plasmid DNA (pDNA) encoding green fluorescent protein EGFP. Since CINVs are characterized by simplified manufacturing technology and a higher yield than natural EVs, as well as a good safety profile (Oshchepkova et al., 2021), we believe that these vesicles may be effective and biocompatible carriers of tNAs.

## 2 Materials and methods

### 2.1 Reagents and materials

Dulbecco’s modified Eagle’s medium (DMEM), Roswell Park Memorial Institute 1640 (RPMI), calcium chloride, chloroquine diphosphate salt (C6628), and sucrose (S0389) were purchased from Sigma. Fetal bovine serum (FBS) was purchased from HyClone. Versene solution was purchased from Biolot. TrypLE™ Express Enzyme, trypan blue solution, sodium pyruvate, and Opti-Minimal Essential Medium (Opti-MEM) were purchased from Gibco. Antibiotic/antimycotic mix (10,000 IU/mL penicillin/10 mg/mL streptomycin/25 μg/mL amphotericin B) and phosphate buffered saline (PBS) were purchased from MP Biomedicals, LCC. Lipofectamine 2000 was purchased from Invitrogen. Cytochalasin B (Cyt B; A7657) was purchased from AppliChem GmbH.

PageRuler™ Plus Prestained Protein Ladder (26619) was purchased from Thermo Scientific. Laemmli buffer (2× concentrate; S3401) and PVDF membrane (IPVH00010) were purchased from Sigma. RIPA buffer and cocktail protease inhibitor (50×) were purchased from Servicebio. Gel-Blotting Paper GB 005 was purchased from GE Healthcare Life Sciences, Whatman. Antibodies used for western blot assays were purchased from Abcam (ab275377, ab52894, ab210546) and ABclonal Inc. (A19524, A19056, A1118, AS014).

6-carboxyfluorescein (FAM) phosphoramidite was purchased from Lumiprobe. TRIzol reagent was purchased from Invitrogen. M-MuLV-RH reverse transcriptase with RT buffer (R03-10) and HS-qPCR SYBR Blue Kit (MHC030-2040) were purchased from Biolabmix. Vector pEGFP-C2 (4,735 bp) was purchased from Clontech.

Cell culture plastic was purchased from TPP. Glass coverslips were purchased from Marienfeld. Western blotting equipment was purchased from Bio-Rad.

### 2.2 Oligonucleotide synthesis and duplex annealing

Strands of isRNA and siRNAs were synthesized on an automatic ASM-800 synthesizer (Biosset) as described previously (Kabilova et al., 2018; Chernikov et al., 2024). An antisense oligonucleotide containing µ-modification of all internucleotidic phosphates was synthesized as described previously (Zharkov et al., 2024). After standard deprotection, the oligonucleotides were purified by 15% denaturing polyacrylamide gel (PAGE) electrophoresis and isolated as sodium salts. Alternately, isolation was performed by reversed-phase high-performance liquid chromatography (HPLC) on an Agilent1260 HPLC system (Agilent Technologies Inc.). The purity of the oligonucleotides was analyzed by a 15% denaturing PAGE. The sequences of the synthesized oligonucleotides are listed in Table 1.

**TABLE 1:** List of synthesized oligonucleotides.

Name	Sequence (5′–3′)
siMDR1, s	GGC​UUG **A** C **AAG** UUG​UAU​AUG​G
siMDR1, as	A **U** AUA **C** AAC​UUG​U **C** A **A** GCC​AA
siMDR1^µ^, s	GμGμCUU​G **A** C **AAG** UUG​UAU​AUμGμG
siMDR1^PS^, s	G*G*CUU​G **A** C **AAG** UUG​UAU​AU*G*G
siMDR1^PS^, as	A*U*AUA **C** AAC​UUG​U **C** A **A** GCC*A*A
isRNA, st 1	AAA​UCU​GAA​AGC​CUG​ACA​CUU​A
isRNA, st 2	GUG​UCA​GGC​UUU​CAG​AUU​UUU​U
siScr, s	CCA​CUA **C** A **UAC** GAG​ACU​UGU​U
siScr, as	C **A** AGU **C** UCG​UAU​G **U** A **G** UGG​UU
isScr, st 1	CCA​CUA​CAU​ACG​AGA​CUU​GUU
isScr, st 2	CAA​GUC​UCG​UAU​GUA​GUG​GUU
μ-ON-21	*T*μ*C*μ*A*μ*A*μ*C*μ*A*μ*T*μ*C*μ*A*μ*G*μ*T*μ*C*μ*T*μ*G*μ*A*μ*T*μ*A*μ*A*μ*G*μ*C*μ*T*μ*A*
FAM-μ-ON-21	FAM-*T*μ*C*μ*A*μ*A*μ*C*μ*A*μ*T*μ*C*μ*A*μ*G*μ*T*μ*C*μ*T*μ*G*μ*A*μ*T*μ*A*μ*A*μ*G*μ*C*μ*T*μ*A*
μ-ON-Scr	*C*μ*A*μ*A*μ*G*μ*T*μ*C*μ*T*μ*C*μ*G*μ*T*μ*A*μ*T*μ*G*μ*T*μ*A*μ*G*μ*T*μ*G*μ*G*μ*T*μ*T*

Scr–scrambled; 2′-F modification is indicated in bold, 2′-O-methyl modification is underlined; μ–mesyl or (methanesulfonyl) modification of internucleotic phosphate; *–phosphorothioate modification; s–sense strand; as–antisense strand; st–strand; ON–antisense oligonucleotide; is–immunostimulatory; deoxyribonucleotide is shown in italics.

The siRNA or isRNA duplexes were obtained via annealing of antisense and sense, or first and second strands, at equimolar concentrations in 15 mM HEPES-KOH (pH 7.4), 50 mM potassium acetate, and 1 mM magnesium acetate. The duplexes were stored at −20°C until use.

### 2.3 Cells

HEK 293, KB-3-1, and K562 cells were purchased from the Institute of Cytology RAS (St. Petersburg, Russia). B16 cells were kindly provided by the National Medical Research Center of Oncology named after N.N. Blokhin (Moscow, Russia). RAW 264.7 cells were provided by Prof. D.V. Kuprash (Engelhardt Institute of Molecular Biology, RAS, Moscow, Russia). KB-3-1-MDR1-GFP and K562-MDR1-GFP cell lines were obtained by transduction of KB-3-1 and K562 cells, respectively, with the lentivirus vector pLVT-MDR1 (299–751 nt)-turboGFPdest1 (Chernikov et al., 2019).

All cell lines were routinely cultured in medium supplemented with 10% FBS and 1% antibiotic/antimycotic mix at 37°C in a humidified atmosphere of 5% CO_2_/95% air. HEK 293, KB-3-1, KB-3-1-MDR1-GFP, and B16 cells were grown in DMEM. K562 and K562-MDR1-GFP cells were grown in RPMI. Raw 264.7 cells were cultured in DMEM (4.5 g/L glucose concentration) supplemented with 1 mM sodium pyruvate.

### 2.4 Preparation of CINVs from live or apoptotic cells

A Cyt B stock solution was prepared in dimethyl sulfoxide, aliquoted, and stored at −20°C. All procedures were performed under sterile conditions. CINVs prepared from late apoptotic/necrotic cells were designated as aCINVs. CINV/aCINV preparation was performed as previously described (Oshchepkova et al., 2019; Oshchepkova et al., 2021) with minor modifications. DMEM was used to prepare CINVs from B16, KB-3-1, or RAW 264.7 cells, and RPMI was used in the case of K562 cells. Adherent (B16, KB-3-1) or semi-adherent (RAW 264.7) cells were grown in 150 or 300 cm^2^ cell culture flasks until 90%–100% confluence. Suspension K562 cells were seeded at a density of 5 × 10^5^ cells/mL in 150 cm^2^ cell culture flasks and pre-incubated in RPMI supplemented with 10% FBS and 50 mM H_2_O_2_ for 24 h to induce cell death (Oshchepkova et al., 2021).

Before CINV/aCINV preparation, B16 or KB-3-1 cells were detached using Versene solution; RAW 264.7 cells were collected in Versene solution using a cell scraper. Collected cells were placed into a 25 cm^2^ cell culture flask with a vented screw cap and incubated in 5 mL of fresh FBS-free medium supplemented with 10 μg/mL Cyt B for 30 min at 37°C and 5% CO_2_/95% air. Then, the flask was vigorously vortexed for 30 s, and CINVs/aCINVs were collected by several consecutive centrifugations (5415R centrifuge; Eppendorf): 100 *g* (10 min, 4°C), 600 *g* (20 min, 4°C, twice), and 15,000 *g* (30 min, 4°C). The pellet obtained after 15,000 *g* was washed twice with 1 mL TBS (20 mM Tris-HCl and 150 mM NaCl, pH 7.4) and once with 1 mL Opti-MEM (15,000 *g*, 30 min, 4°C). The CINV/aCINV pellet was re-suspended in Opti-MEM and stored at −80°C until use. The yield of CINVs/aCINVs was evaluated by measuring the total protein concentration.

The following abbreviations were used to designate CINVs/aCINVs prepared from cells of different origins: from B16 cells–B16 CINVs; from KB-3-1 cells–KB CINVs; from late apoptotic/necrotic K562 cells–K562 aCINVs; and from RAW 264.7 cells–RAW CINVs.

### 2.5 Measurement of the total protein concentration

The Qubit™ protein assay kit (Invitrogen) or QuDye protein quantification kit (Lumiprobe) was used to assay the total protein concentration in CINV/aCINV preparations using a Qubit 2.0 fluorimeter. Samples were lysed in 0.5% sodium dodecyl sulfate (SDS) for 15 min at room temperature. Fluorescence was measured at 485/510–580 nm. The dilution used for the sample measurements was 1:1000.

### 2.6 Cell counting and viability assay

Cell number and viability were determined using an automated cell counter, TC-20 (Bio-Rad), and a 0.4% trypan blue solution. Experiments were performed under antibiotic/antimycotic-free conditions and repeated at least twice. Cells were detached before measurements by TrypLE™ Express Enzyme. In experiments with CINVs/aCINVs, cells were incubated in the presence of 20, 50, or 100 µg per well (total protein) of CINVs/aCINVs unloaded with tNAs in the appropriate medium supplemented with 10% FBS depleted from EVs (EV-depleted FBS) for 24 or 72 h. CINVs/aCINVs were added to cells in 20 µL Opti-MEM, and 20 µL Opti-MEM was added to control cells. EV-depleted FBS was prepared by overnight centrifugation at 100,000 *g* (Beckman coulter, Avanti J-30I, JA 30.50 Ti rotor).

### 2.7 Western blot analysis

One million cells per well were seeded in a 6-well plate ∼17 h before the experiments. On the day of the experiment, the plate was placed on ice, and the cells were washed twice with cold PBS and incubated for 5 min with 250 µL RIPA buffer (50 mM Tris-HCl pH 7.4, 150 mM NaCl, 1 mM EDTA-2Na, 1% Triton X-100, 1% sodium deoxycholic acid, 0.1% SDS) supplemented with protease inhibitor cocktail. The obtained lysates were collected and placed into 1.5-mL tubes. The samples were shaken at 400 rpm for 30 min (4°C), followed by centrifugation at 12,000 rpm for 20 min (4°C). The supernatants were collected and mixed with Laemmli buffer (4% SDS, 20% glycerol, 10% 2-mercaptoethanol, 0.004% bromphenol blue, and 0.125 M Tris HCl, pH ∼6.8.) (1:1, vol.). The samples were heated at 95°C (10 min), frozen in liquid nitrogen, and stored at −80°C until analysis. Lysis of CINVs/aCINVs was performed either in a manner similar to that in cells or directly in Laemmli buffer (1:1, vol.) supplemented with a protease inhibitor cocktail.

Gel electrophoresis was performed using 10% SDS-PAGE for 1.5–2 h. Running buffer contained 25 mM Tris base, 190 mM glycine, and 0.1% SDS (pH 8.3). Transfer of proteins (wet) to the PVDF membrane was performed in the same buffer supplemented with 20% ethanol. The transfer was performed for 2 h at 270 mA.

The PVDF membrane was blocked for 1 h at room temperature using blocking buffer containing TBS (pH 7.2) supplemented with 2% skim milk and 0.1% Tween-20. The primary antibody was diluted in blocking buffer and incubated with the membrane overnight at 4°C with constant agitation at 180 rpm. The HRP-conjugated secondary antibody was diluted in blocking buffer and incubated with the membrane for 1 h at room temperature with constant agitation at 180 rpm. The chemiluminescence kit (Servicebio) was used according to the manufacturer’s recommendations, and proteins were immediately analyzed using an iBright 1500 imaging system (Invitrogen). Antibodies were used according to the manufacturer’s recommendations.

### 2.8 Preparation of 2X3-DOPE liposomes

Cationic liposomes containing 1,26-bis(cholest-5-en-3β-yloxycarbonylamino)-7,11,16,20-tetraazahexacosane tetrahydrochloride (2X3) (Petukhov et al., 2010) and the helper lipid 1,2-dioleoyl-*sn*-glycero-3-phosphoethanolamine (DOPE; Lipoid) were prepared by hydrating of thin lipid film as described previously (Luneva et al., 2018). The final liposome concentration was 1 mM.

### 2.9 Loading of CINVs/aCINVs with tNAs by freezing-thawing (Fr-Th)

The loading mixture was prepared in 20 µL Opti-MEM for less than 200 µg CINVs/aCINVs or in 50 µL Opti-MEM for *≥* 200 µg CINVs. The loading of CINVs/aCINVs was performed by three rounds of sample freezing in liquid nitrogen, followed by thawing (Oshchepkova et al., 2019; Oshchepkova et al., 2021). Samples containing CINVs/aCINVs and tNAs were frozen and kept at −80°C for 10 min. Thawing was performed in a water bath at room temperature, followed by vigorous shaking at 700 rpm (25°C) for 10 min. After the third freezing step, samples were left at −80°C overnight, thawed the next day, shaken at 700 rpm (25°C) for 10 min, and added to cells. The Fr-Th approach was used in all cases unless otherwise indicated in the text or figure legends.

### 2.10 Loading of CINVs with siRNA by chemical permeabilization (CaCl_2_ method)

Twenty micrograms of KB CINVs were mixed with 0.05 or 1.0 nmol siMDR1 or 0.05 nmol siScr in 20 µL Opti-MEM supplemented with CaCl_2_ at a final concentration of 0.1 M (Zhang et al., 2017). Samples were placed on ice and incubated for 30 min. Heat shock was performed at 42°C for 60 s, followed by 5 min of incubation on ice. The volume in tubes was increased to 50 µL by Opti-MEM, and the samples were centrifuged at 15,000 *g* (30 min, 4°C). The pellet was suspended in 20 µL Opti-MEM and added to the cells.

### 2.11 Pre-complexing of tNAs with 2X3-DOPE liposomes or Lipofectamine 2000

Complexes of tNAs and Lipofectamine 2000 (LF) were prepared according to the manufacturer’s recommendations. Lipoplexes of tNAs with 2X3-DOPE liposomes were prepared in 50 µL Opti-MEM for *in vitro* experiments and in 200 µL Opti-MEM for *in vivo* experiments. 2X3-DOPE liposomes were incubated in 25 or 100 µL Opti-MEM for 5 min at room temperature. Then, an equal volume of tNA in Opti-MEM was added to the liposome solution. The resulting mixture was incubated for 20 min at room temperature before use. The concentrations of 2X3-DOPE liposomes in the mixture correspond to different N/P ratios (the ratio of positively charged amine (N) groups in the liposomes to negatively charged phosphate (P) groups in tNAs). Lipoplexes of tNAs/2X3-DOPE were formed at the N/P ratio 4/1 for siRNA, isRNA, and ASO and at the N/P ratio 10/1 in the case of pDNA.

### 2.12 CINV/aCINV-mediated tNA delivery: *In vitro* experiments

All experiments were performed in antibiotic/antimycotic-free medium. KB-3-1, KB-3-1-MDR1-GFP, HEK 293, and B16 cells were seeded in a 48-well plate at a density of 23.5 × 10^3^ cells per well in DMEM supplemented with 10% EV-depleted FBS (250 µL) for 17–24 h before the experiments. B16 cells were seeded in a 24-well plate (8 × 10^4^ cells per well) for ASO delivery or in a 96-well plate (3 × 10^3^ cells per well) for experiments with isRNA in 500 or 150 µL of the same medium, respectively. Suspension K562-MDR1-GFP cells were seeded on the day of the experiment at a density 4.5 × 10^4^ cells per well in a 48-well plate in 250 µL RPMI supplemented with 10% EV-depleted FBS.

The experimental conditions used for the delivery of various tNAs by CINVs/aCINVs are summarized in Table 2. Delivery of tNAs by CINVs/aCINVs or 2X3-DOPE liposomes was performed in the presence of 10% EV-depleted FBS. In contrast, LF-mediated delivery of tNAs was performed under FBS-free conditions. In the experiments with siRNA, isRNA, and pDNA, cells were incubated with loaded vesicles, and the volume of the cell medium was not changed during the experiments. In the case of ASO delivery, B16 melanoma cells were incubated with B16 CINVs in a total volume of 200 µL/well of a 24-well plate for 4 h, then 300 µL per well of the same medium was added, followed by incubation for another 68 h. The 120-h incubation of KB-3-1 cells with KB CINVs loaded with pDNA was carried out as follows: after 72 h, 1/4 of the cells were re-plated and cultured for an additional 48 h. The final volume in a well was 150 µL in a 96-well plate, 250 µL in a 48-well plate, and 500 µL in a 24-well plate.

**TABLE 2:** Conditions for tNA delivery by CINVs/aCINVs.

tNAs	Cells	CINVs/aCINVs	Amount in the loading mixture		Time of incubation, h	
			tNAs,nmol/µg^e^	CINVs/aCINVs, µg	With tNAs^a^	Total
ASO	B16	B16	0.02	50	72	72
		B16	0.05	200	24^d^	24
siRNA	KB-3-1-MDR1-GFP	KB	0.05, 1.0	20	72	72
		KB	0.05, 1.0	20	4^b^	72
		KB	0.05	100	72	72
	K562-MDR1-GFP	K562	0.05, 1.0	20	72	72
isRNA	B16	B16 or RAW	0.015	100	72	72
pDNA	KB-3-1	KB	0.25^e^, 1.5^e^	20	72	72
		KB	0.25^e^	100	72	72
		KB	0.25^e^	20,100	72	120
		KB	0.25^e^	100	28^c^	72
	B16	B16	0.25^e^	100	72	72
		B16 or RAW	0.25^e^	200	72	72
	HEK 293	B16	0.25^e^	100	72	72

^a^ loaded into CINVs/aCINVs.

^b^ experiments with 0.2 M sucrose.

^c^experiments with chloroquine.

^d^ confocal microscopy assay.

e µg.

Incubation of KB-3-1-MDR1-GFP cells with KB CINVs loaded with siRNA in the presence of 0.2 M sucrose was performed for 4 h. KB-3-1 cells were incubated with 50 µM chloroquine as follows: complexes of pDNA with KB CINVs (pDNA/KB CINVs) were incubated with cells for 4 h, after which chloroquine was added at a final concentration of 50 µM for 24 h. A stock solution of chloroquine was prepared in Opti-MEM on the day of the experiments.

Delivery of tNAs by LF or 2X3-DOPE liposomes was used as a positive control and performed as previously described (Maslov et al., 2012; Miroshnichenko et al., 2019; Patutina et al., 2020). A similar amount of tNAs (see Table 2) was pre-complexed with LF or 2X3-DOPE liposomes as described in Section 2.11 and added to cells. After 4 h of incubation with lipoplexes, the medium was replaced with a fresh portion supplemented with 10% EV-depleted FBS, and cells were incubated for an additional 68 h, except for longer incubation with pDNA (120 h) or shorter incubation with ASO (24 h), similar to the conditions used for experiments with CINVs/aCINVs.

Independent experiments with siRNA and pDNA were performed at least twice. Experiments with ASO were performed three times. The delivery of isRNA by B16 CINVs or RAW CINVs was performed four and three times, respectively.

### 2.13 CINV-mediated isRNA delivery: *in vivo* experiment

Female 10- to 14-week-old CBA/LacSto (hereafter, CBA) mice with an average weight of 19.5–22 g were obtained from the vivarium of the Institute of Chemical Biology and Fundamental Medicine SB RAS, Novosibirsk, Russia. Mice were housed in plastic cages under normal daylight conditions. Water and food were provided *ad libitum*.

RAW CINV (200 μg) loading with isRNA (10 μg) was performed by Fr-Th as described in Section 2.9. Before injection, the volume of each sample was adjusted to 200 µL with Opti-MEM. Lipoplexes of isRNA (10 μg per mouse) with 2X3-DOPE liposomes were formed at the N/P ratio 4/1 as described in Section 2.11 in a total volume of 200 µL.

The complexes of isRNA/RAW CINVs or isRNA/2X3-DOPE were injected intravenously (i.v.) into the tail vein of CBA mice in 200 µL Opti-MEM. After 6 h, peripheral blood was collected from the retro-orbital sinus. Blood serum was prepared from whole blood by clot coagulation at 37°C for 30 min and 4°C overnight, followed by centrifugation at 4,000 rpm (4°C, 20 min). Serum samples were used to measure the level of interferon-alpha (IFN-α) using the IFN-alpha ELISA kit (Invitrogen). Absorbance was measured at 450 nm using a Multiskan RC reader (Thermo Labsystems); measurements were performed in duplicate. Each experimental group consisted of three mice. Control mice received the i.v. injection of 200 µL Opti-MEM.

### 2.14 Electrophoretic mobility shift (gel shift) assay

Experiments using native 12% PAGE were performed in TBE (45 mM Tris, 45 mM boric acid, 1 mM EDTA, pH 8.3) as the running buffer. In particular, 20, 40, 80, or 160 μg KB CINVs were loaded with 0.05 nmol siMDR1 in 20 µL Opti-MEM by Fr-Th (see Section 2.9). Twelve microliters of 20% Ficoll (aqueous solution) were added to each sample before loading on the gel. Electrophoresis was performed at 400 V for ∼2 h at 4°C. The gel was stained with 0.1% Stains-All (Sigma) for 12–15 min at room temperature.

KB CINVs (20, 50, or 100 µg) loaded with either 0.05 nmol siMDR1 or 0.25 µg pDNA and 20, 50, or 100 µg B16 CINVs loaded with 0.02 nmol μ-ON-21 by Fr-Th in 20 µL Opti-MEM were applied on 4% (1% for pDNA-loaded CINVs) agarose gel containing 5 μg/mL ethidium bromide in TAE (40 mM Tris, 20 mM acetate, and 1 mM EDTA, pH 8.6) buffer (Huang et al., 2015). Before loading on the gel, the complexes of tNAs/CINVs were supplemented with glycerol at a final concentration of 5%. Electrophoresis was performed at 120 V for 35–40 min (room temperature).

All images of the gels were made using the iBright 1500 imaging system.

### 2.15 Flow cytometry assay

Flow cytometry measurements were performed using a NovoCyte™ flow cytometer (ACEA Biosciences, Inc.). The following gating strategy was used: SSC-H vs FSC-H to identify cells by granularity/size and FSC-H vs FSC-A to exclude cell doublets. Cells were analyzed immediately after the experiments, without fixation. The procedure of cell detachment was performed on ice using TrypLE™ Express Enzyme.

The levels of MDR1-GFP or EGFP protein were analyzed in the fluorescein isothiocyanate (FITC, excitation–488 nm; detection–530 ± 30 nm) channel. For convenience, data measured in relative fluorescence units (RFU) are presented in the figures as mean fluorescence intensities of cells in a population divided by 1000.

### 2.16 Stem-loop reverse transcription qPCR

The levels of microRNA-21 (miR-21) and let-7g in B16 cells were estimated using stem-loop RT-qPCR (Chen et al., 2005; Varkonyi-Gasic et al., 2007). Total RNA was isolated from cells using TRIzol reagent according to the manufacturer’s recommendations. The reaction mix (20 µL) containing RT-buffer (50 mM Tris–HCl (pH 8.3), 50 mM KCl, 4 mM MgCl_2_, 0.5 mM dNTP, 10 mM DTT), 100 U M-MuLV-RH reverse transcriptase, 3 µg total RNA, and 50 nM specific primers was incubated as follows: 16°С for 30 min (1 cycle); 30°С for 30 s, 42°С for 30 s, 50°С for 30 s (60 cycles). To terminate the reaction, reverse transcriptase was inactivated at 85°С for 5 min. Primers for reverse transcription (from 5′to 3′) were as follows: RT-miR-21 GTC​GTA​TCC​AGT​GCA​GGG​TCC​GAG​GTA​TTC​GCA​CTG​GAT​ACG​ACTCA​ACA​TCA​G; RT-let-7g GTC​GTA​TCC​AGT​GCA​GGG​TCC​GAG​GTA​TTC​GCA​CTG​GAT​ACG​ACAAC​TGT​ACA​A; RT-Gapdh GGC​ATG​GAC​TGT​GGT​CAT​GAG; and RT-Hprt1 AAC​AAA​GTC​TGG​CCT​GTA​TCC. The sequence complementary to the 3′-end of miRNA or housekeeping gene mRNA is underlined.

PCR was performed in a reaction mix (20 µL) containing PCR-buffer (50 mM Tris–HCl (pH 8.5), 50 mM KCl, 1.5 mM MgCl_2_, 0.2 mM of each dNTP, 0.03 U Taq-Polymerase, 0.0125% Tween-20, SYBR Green I), 5 µL cDNA (10:1 dilution), 0.25 µM of specific primers (see below) as follows: 95°С for 4 min (1 cycle); 95°С for 40 s, 60°С for 30 s, 72°С for 30 s (40 cycles). Primer sequences (from 5′ to 3′) were as follows: miR-21-F AGA​CTA​GCT​TAT​CAG​ACT​GA; let-7g-F AAC​GCT​GAG​GTA​GTA​GTT​TGT; Universal reverse primer GTGCAGGGTCCGAGGT; Gapdh-F TGC​ACC​ACC​AAC​TGC​TTA​GC; Gapdh-R GGC​ATG​GAC​TGT​GGT​CAT​GA; Hprt1-F CCC​CAA​AAT​GGT​TAA​GGT​TGC; Hprt1-R AAC​AAA​GTC​TGG​CCT​GTA​TCC.

The melting curve data were obtained with a 0.5°С interval, starting from 55°С to 95°С. The obtained qPCR data were analyzed using the standard Bio-Rad iQ5 v.2.0 software. The ΔΔCt method was used to determine the relative miRNA levels with *Gapdh* and *Hprt1* serving for normalization.

### 2.17 WST-1 assay

The anti-proliferative effect of isRNA in B16 cells was estimated using the WST-1 cell proliferation assay kit (Takara Bio Inc. or Roche). The WST-1 assay was performed according to the manufacturer’s recommendations. Briefly, cells were incubated with isRNA loaded into CINVs or complexed with 2X3-DOPE liposomes, and WST-1 solution was added to each well at a dilution of 1:10 (vol.) and incubated with the cells for 30 min at 37°C and 5% CO_2_/95% air (Bishani et al., 2023). Absorbance was measured using the Multiskan RC reader at 450 nm, and 620 nm was used as the reference wavelength.

### 2.18 Confocal microscopy

B16 cells were plated on glass coverslips in a 24-well plate. Cells were incubated for 24 h with either (1) 200 µg B16 CINVs loaded with 0.05 nmol FAM-μ-ON-21 (Table 1 and Table 2) by Fr-Th or (2) 200 µg unloaded B16 CINVs, subjected to Fr-Th procedures, and stained with the CellTrace™ CFSE Cell Proliferation Kit (Invitrogen). After 24 h of incubation, the coverslips with cells were washed twice with PBS, fixed with 4% formaldehyde (Central Drug House (P) Ltd.) in PBS for 15 min at 37°C, and washed twice with PBS. The actin filaments of the cells were stained with Phalloidin-iFluor 647 reagent (Abcam) according to the manufacturer’s recommendations. The cells were then washed twice with PBS, and the nuclei were stained with 10 μg/mL DAPI (62248; Thermo Scientific) solution in PBS for 10 min at room temperature. The coverslips were mounted on the slide using Fluoromount-G^®^ (SouthernBiotech) and incubated for 12–18 h at 25°C in the dark on a flat, dry surface.

Staining of B16 CINVs with the CellTrace™ CFSE Cell Proliferation Kit (5 µM CellTrace™ CFSE) was performed in 200 µL Opti-MEM for 20 min at 25°C and constant shaking at 300 rpm, followed by the addition of 10% EV-depleted FBS and further incubation of CINVs for 5 min under the same conditions. The CFSE-labeled B16 CINVs were pelleted at 15,000 g (30 min, 4°C), resuspended in 50 µL Opti-MEM, and added to the cells.

The intracellular localization of FAM-μ-ON-21/B16 CINVs or CFSE-labeled B16 CINVs was analyzed by confocal scanning microscopy on an LSM710 microscope (Zeiss) using a Plan-Apochromat 63x/1.40 Oil DIC M27 objective and ZEN Black software (Zeiss). The confocal microscopy assay was performed in three channels (blue, green, and red). Fluorescence in the blue channel corresponded to DAPI (nuclear staining); the green channel corresponded to fluorescence of FAM-μ-ON-21 or CFSE-labeled B16 CINVs; and the red channel corresponded to Phalloidin-iFluor 647 (cytoskeleton staining). The experiments were repeated twice and performed in duplicate.

### 2.19 Statistical analysis

Data are presented as mean and standard deviation (SD) or standard error (SE). Pairwise comparisons were performed using the non-parametric Mann–Whitney *U*-test.

## 3 Results

Natural EVs and their mimetics are particularly promising tNA carriers because of their high biocompatibility, potential for tissue-specific accumulation, and ability to transfer biologically active cargo between cells. This study employed cell treatment with Cyt B to generate EV mimetics (CINVs/aCINVs) to deliver four types of tNAs. CINVs/aCINVs were prepared from B16 mouse melanoma cells (B16 CINVs), KB-3-1 human carcinoma cells (KB CINVs), late apoptotic/necrotic K562 human myelogenous leukemia cells (K562 aCINVs), and RAW 264.7 mouse macrophage-like cells (RAW CINVs). These vesicles have been successfully used previously to deliver a FAM-labeled DNA oligonucleotide to various cells *in vitro* (Oshchepkova et al., 2021). Lipofectamine 2000 (LF) and cationic liposomes 2X3-DOPE were used for comparison.

The current study focused on the CINV-mediated delivery of the most prevalent types of tNAs used in clinical practice: antisense oligonucleotide (ASO), small interfering RNA (siRNA), immunostimulatory RNA (isRNA), and transgene-expressing construct–plasmid DNA. In the first step, the ability of CINVs to deliver ASO containing µ-modifications of all internucleotidic phosphates and exhibiting record nuclease resistance and RNase H compatibility (Miroshnichenko et al., 2019) was examined. ASO was targeted to a multifunctional oncogenic microRNA-21 (µ-ON-21; Table 1), a promising therapeutic target for multiple types of malignancies (Bautista-Sánchez et al., 2020; Gaponova et al., 2022; Hashemi et al., 2023). The studied siRNA was a highly nuclease-resistant RNA duplex containing 2′-O-methyl (2′-OMe) and 2′-F modifications (Table 1) and silencing the *MDR1* gene *in vitro* and *in vivo* (Chernikov et al., 2023; Chernikov et al., 2024). The isRNA (Table 1) was a short 22-bp RNA duplex containing no chemical modifications and having no homology with the human and mouse genomes. It exhibited significant anti-proliferative activity against several tumor cells *in vitro* and stimulated synthesis of interferon-alpha (IFN-α) *in vivo* (Kabilova et al., 2016; Bishani et al., 2023). Finally, the delivery of augmentation gene therapy agents was modeled using the pEGFP-C2 plasmid encoding the EGFP protein.

### 3.1 Characterization of CINVs/aCINVs

**FIGURE 1:**
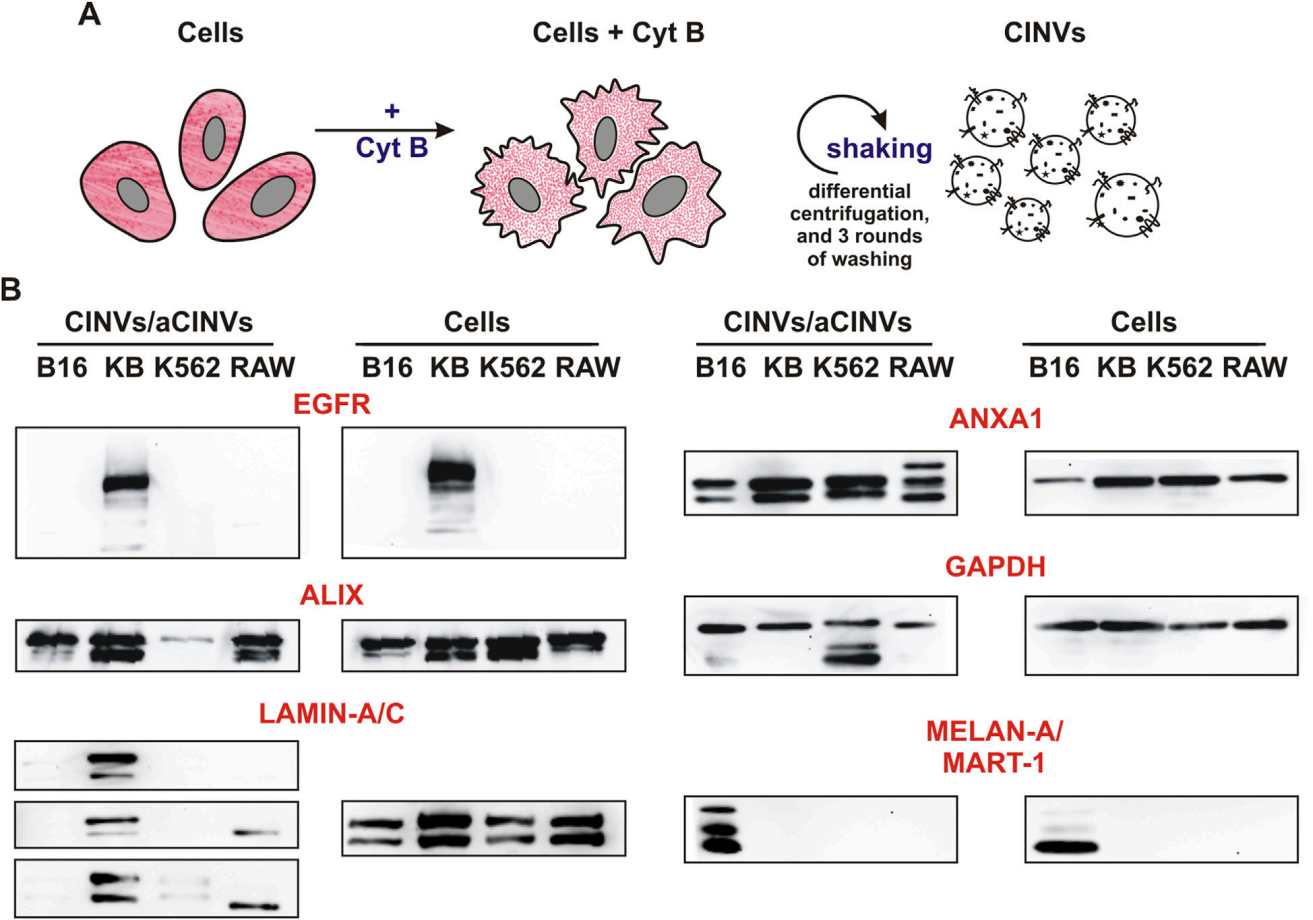
Isolation and characterization of CINVs/aCINVs. **(A)** Scheme of CINV/aCINV preparation. Cells were incubated with 10 μg/mL Cyt B for 30 min to destroy actin filaments. Since the plasma membrane loses its structure in the absence of a normal actin network, mechanical shaking of Cyt B-treated cells causes the shedding of vesicles from their surface. **(B)** Western blot assay of CINVs/aCINVs (n = 3). The names of the proteins are shown for humans. GAPDH was used as the loading control. Live cells were used to prepare cell lysates and CINVs, with the exception of K562 aCINVs, which were generated from late apoptotic/necrotic K562 cells. The staining of CINVs/aCINVs by anti-LAMIN-A/C antibodies was characterized by high variability; therefore, the results of three experiments were presented.

According to our previous observations (Oshchepkova et al., 2019; Oshchepkova et al., 2021), the current procedure of CINV/aCINV isolation (Figure 1A) allows the production of vesicles of 100–150 nm in diameter, which are suitable for the delivery of tNAs. Here, the obtained CINVs/aCINVs were characterized by several proteins using the western blot assay (Figure 1B; all proteins in the text were indicated for humans).

First, we observed that CINVs/aCINVs contained tissue-specific markers of their parent cells. In particular, KB CINVs were enriched with epidermal growth factor receptor (EGFR) similar to KB-3-1 cells, and B16 CINVs were positive for melanoma-specific antigen (MELAN-A/MART-1) (Figure 1B). Similar to natural EVs (Pessolano et al., 2019; Perez et al., 2023), CINVs/aCINVs were enriched with the ANXA1 protein (Figure 1B), which was present in both full-length and cleaved forms. According to earlier observations, ANXA1 may serve as a specific marker for EVs secreted by shedding from the plasma membrane (microvesicles) (Jeppesen et al., 2019). CINVs/aCINVs are considered mimetics of microvesicles because they are secreted by cells in a similar manner. ALIX is a multifunctional protein that is often referred to as an exosomal marker (Jeppesen et al., 2019). Nonetheless, ALIX can be found in the cytoplasm of cells, so it was also found in CINVs/aCINVs (Figure 1B). The nuclear envelope protein LAMIN-A/C was assayed in CINVs/aCINVs to analyze their contamination with cell nuclei. Overall, LAMIN-A/C was consistently found only in KB CINVs (Figure 1B), indicating that the current protocol for CINV/aCINV isolation was suitable for initial laboratory experiments but should be improved to obtain more pure preparations.

At the next stage, we tested the influence of CINVs/aCINVs on cell survival and proliferation because a residual amount of Cyt B was previously found in CINV preparations (Oshchepkova et al., 2021). Since Cyt B can induce cell apoptosis, the concentration of CINVs/aCINVs must be optimized to ensure their safe use. Here, we modified the protocol for CINV/aCINV preparations by increasing the number of washing steps from 1 to 3 (Figure 1A). The observed minimal, if any, impact of CINVs/aCINVs on cell survival (Supplementary Figure S1) indicates satisfactory experimental conditions (Table 2). On the contrary, in some cases, an increase in cell counts was observed after treatment with CINVs/aCINVs (Supplementary Figure S1). Considering that Cyt B in low doses can stimulate cell proliferation (Oshchepkova et al., 2021), we hypothesize that the CINV/aCINV preparations still contain a residual but safe amount of Cyt B.

### 3.2 Delivery of ASO by CINVs

**FIGURE 2:**
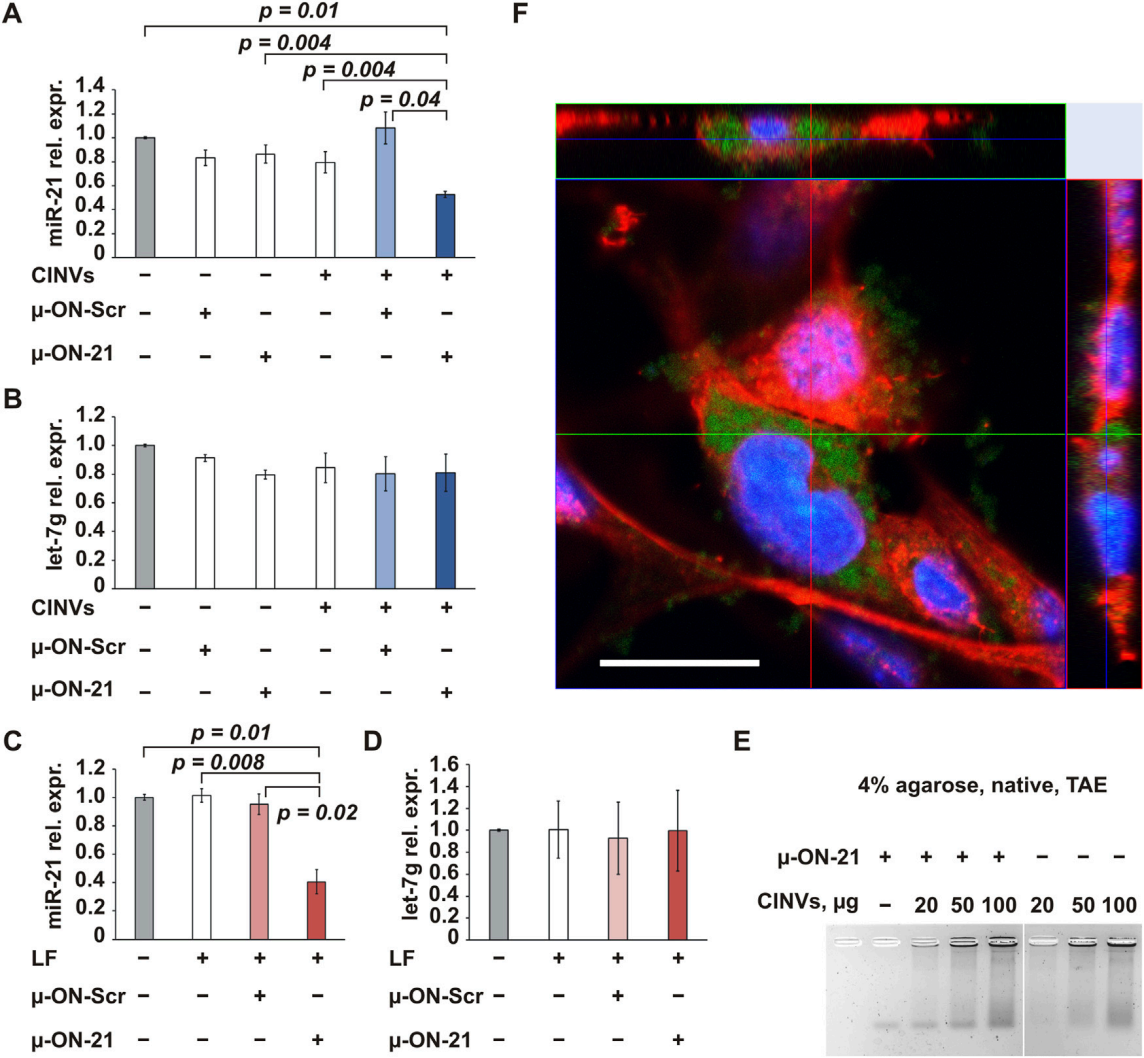
CINV-mediated delivery of ASO. The delivery of µ-ON-21 to B16 cells by B16 CINVs (50 µg/well) **(A, B)** or LF **(C, D)**. The relative expression (rel. expr.) of miR-21 or let-7g miRNAs was assessed by stem-loop RT-qPCR; the expression of miRNAs was normalized to housekeeping genes *Hprt1* and *Gapdh* (n = 3–7). Data are presented as mean and standard error (SE). **(E)** Complexes of µ-ON-21/B16 CINVs were analyzed by the gel shift assay (n = 2–4). **(F)** The intracellular localization of FAM-labeled µ-ON-21 (FAM-µ-ON-21) in B16 cells after CINV-mediated delivery was confirmed by the confocal microscopy assay (Z-stack image, scale bar = 20 µm). The nuclei are indicated by blue color, actin filaments–by red color, and FAM-µ-ON-21 is indicated by green signal (n = 2).

The first type of tNA investigated in this study was 22-nt mesyl (µ) ASO targeted to pro-oncogenic microRNA-21 (Table 1; µ-ON-21). The delivery of µ-ON-21 by B16 CINVs was analyzed in B16 cells by measuring the level of miR-21 using stem-loop RT-qPCR (Figure 2A). We observed a twofold reduction in miR-21 levels in cells after both CINV- and LF-mediated delivery (Figures 2A, C). Moreover, the activity of µ-ON-21/B16 CINVs was specific since the level of miR-21 did not change after the delivery of µ-ON-Scr (Figure 2A), and µ-ON-21 did not affect the level of let-7g miRNA (Figure 2B) in a manner similar to that observed with LF (Figures 2C, D). It is worth mentioning that the transfection efficiency of B16 CINVs and LF was similar: we observed a ∼60% decrease in the level of miR-21 in B16 cells after the delivery of µ-ON-21 by B16 CINVs or LF (Figures 2A, C). It is noteworthy that we have previously found that CINVs and LF can be equally effective for the delivery of a FAM-labeled oligodeoxyribonucleotide (Oshchepkova et al., 2019). It was interesting that the complexes of µ-ON-21/B16 CINVs were not detected using the gel shift assay (Figure 2E).

**FIGURE 3:**
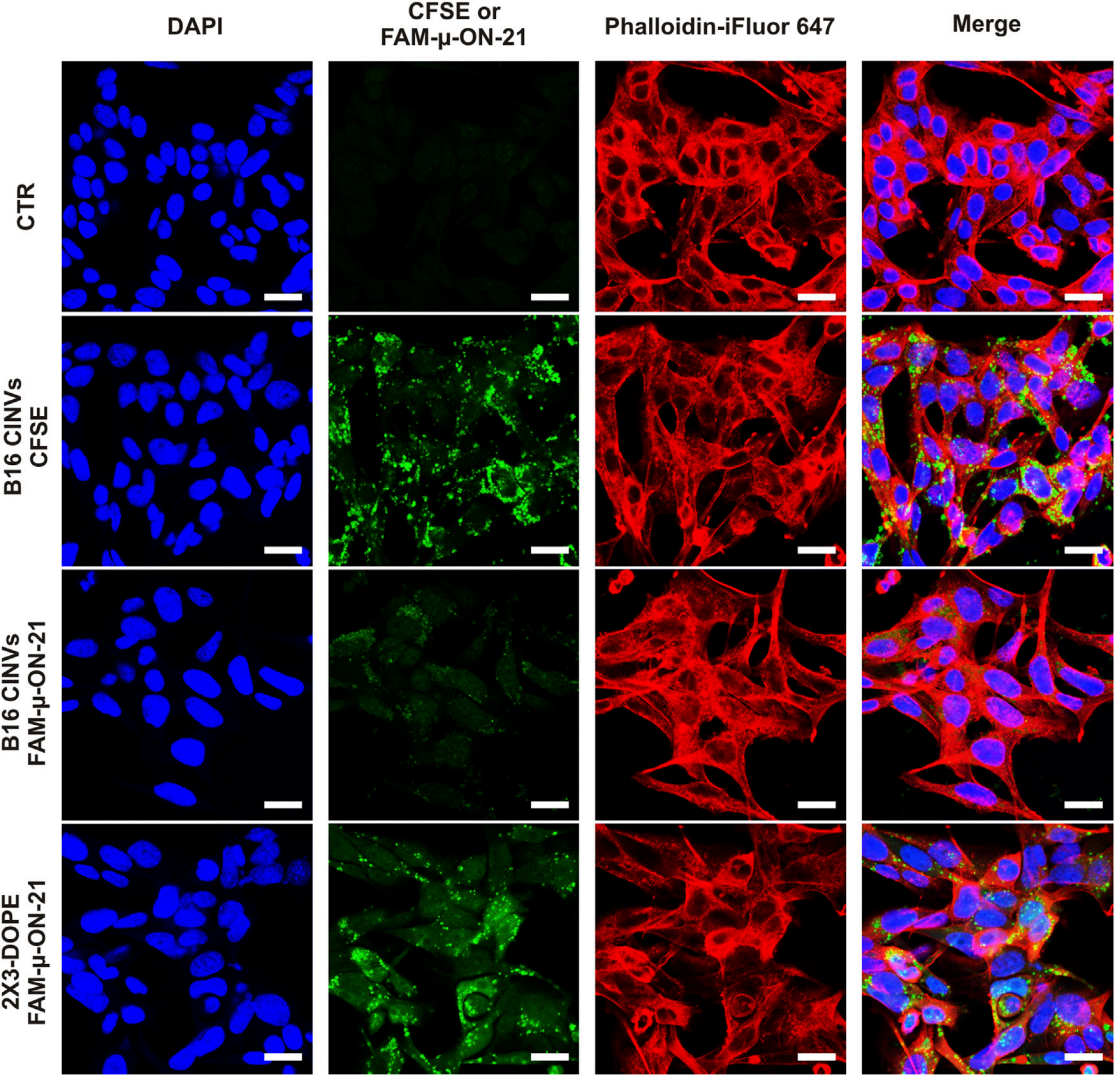
Confocal microscopy analysis of B16 cells incubated with B16 CINVs either loaded with FAM-µ-ON-21 or stained with CFSE dye. CTR–control untreated cells. The nuclei are indicated by blue color, actin filaments–by red color; CFSE or FAM-µ-ON-21 is indicated as green signal. All images in the “green” channel were made with identical settings. The scale bar is 20 µm.

To confirm localization of µ-ON-21 inside B16 cells, FAM-labeled µ-ON-21 (FAM-µ-ON-21) was delivered into the cells by B16 CINVs, followed by a confocal microscopy assay. After 24 h of cell incubation with the loaded CINVs, the FAM-µ-ON-21 was found in the cytoplasm of the recipient cells (Figures 2F, 3). Moreover, B16 CINVs retained their integrity during this time and were found on the surface and inside of B16 cells in great abundance (Figure 3; Supplementary Figure S2A; CFSE staining). The data presented in Supplementary Figure S3 provide evidence that neither the self-penetration of FAM-µ-ON-21 nor the unbound CFSE dye is responsible for the obtained results.

### 3.3 Delivery of siRNA by CINVs/aCINVs

Another type of tNA examined in this study was 2′-OMe, 2′-F fully modified anti-*MDR1* siRNA (Table 1; siMDR1). The *MDR1* gene encodes the P-glycoprotein, whose overexpression leads to tumor cell resistance to chemotherapeutics (Gottesman et al., 1995; Robey et al., 2018). Inhibition of *MDR1* can restore cell sensitivity and enhance antitumor therapy. The MDR1-GFP cell lines contained a fragment of *MDR1* mRNA fused with the mRNA of a short-lived turboGFP and were used to test the functionality of siMDR1 delivery. The level of MDR1 depletion was assessed by measuring GFP fluorescence using flow cytometry.

**FIGURE 4:**
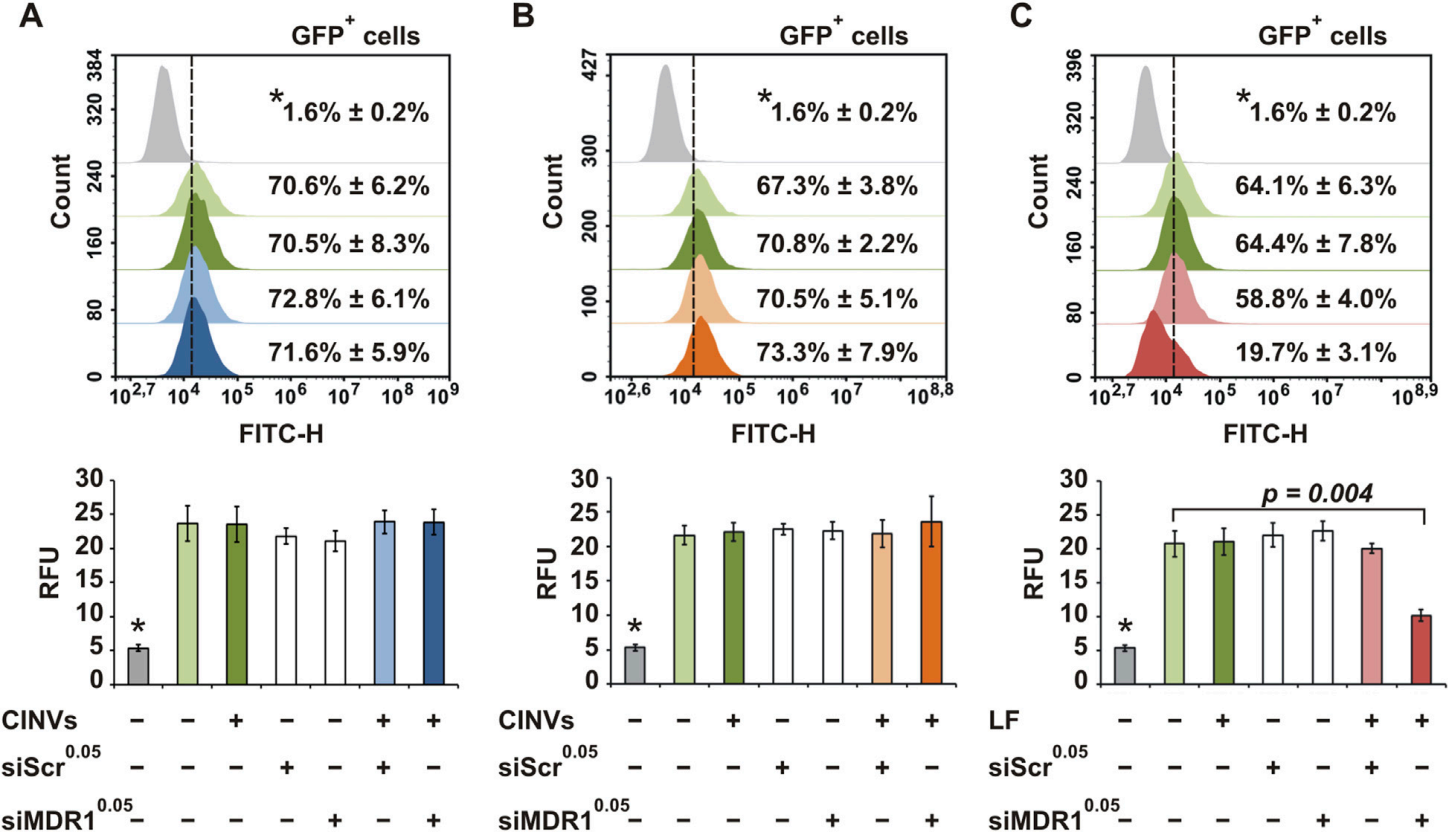
Delivery of siMDR1 to KB-3-1-MDR1-GFP cells. Flow cytometry assay data. KB CINVs (20 µg/well) were loaded with siMDR1 either by Fr-Th **(A)** or chemical permeabilization **(B)**. **(C)** LF-mediated delivery of siMDR1. Summary fluorescence data are presented as percentage of GFP-positive cells (top histograms; count vs FITC-H) and relative fluorescence units (bottom graphs; RFU). The superscript indicates the amount (nmol) of siMDR1 or siScr (scrambled) used in the loading mixture. Parental KB-3-1 cells that do not express GFP are indicated by an asterisk (*). The number of measurements of KB-3-1 cells (*) was 3; in other groups, it varied from 4 to 8. Data are presented as mean and standard deviation (SD).

KB CINVs were loaded with siMDR1 by Fr-Th and added to KB-3-1-MDR1-GFP cells for 72 h. We found that siMDR1 did not decrease the GFP signal in KB-3-1-MDR1-GFP cells, indicating either the absence of intracellular accumulation or non-functional delivery (Figure 4A). A 20-fold increase in the amount of siMDR1 in the loading mixture also showed no decrease in the GFP signal (Supplementary Figure S4A; siMDR1^1.0^). The term “loading mixture” refers to the step in which nucleic acids and CINVs/aCINVs are mixed and subjected to loading procedures (see Sections 2.9–2.10 for details). Similar to Fr-Th, siMDR1 loading into KB CINVs using chemical permeabilization (Figure 4B; Supplementary Figure S4B) did not lead to functional delivery. In contrast, delivery of siMDR1 by LF provided an efficient decrease in the MDR1-GFP signal (Figure 4C), highlighting the problem of using CINVs as siRNA carriers.

The delivery of siMDR1 by K562 aCINVs to K562-MDR1-GFP cells was also unsuccessful (Supplementary Figure S5A). Moreover, the addition of a lysosomo/endosomo-tropic agent (0.2 M sucrose) (Ciftci and Levy, 2001; Caron et al., 2004) had no effect on siMDR1 silencing activity (Supplementary Figure S4C), indicating that siMDR1 was not trapped in lysosomes or endosomes. Neither KB CINVs nor Cyt B prevented siMDR1 action when LF was used as the transfection agent (Supplementary Figure S6), allowing us to rule out the possibility that CINVs or Cyt B inhibited siRNA activity. Moreover, we examined the KB CINV-mediated delivery of siMDR1, which had been additionally stabilized by phosphorothioate (siMDR1^PS^) or µ (siMDR1^µ^) modifications (Supplementary Figure S7A; Table 1) or increased the amount of KB CINVs in the loading mixture fivefold (Supplementary Figure S7C; 100 µg/well KB CINVs). However, none of these conditions resulted in the functional delivery of siRNA in KB-3-1-MDR1-GFP cells.

We examined the electrophoretic mobility of siMDR1-loaded KB CINVs on native 12% polyacrylamide (Supplementary Figure S8A) or 4% agarose (Supplementary Figure S8B) gels and did not observe the formation of siMDR1/KB CINV complexes in either case. Apparently, the complexes of siMDR1/KB CINVs were either unstable under electrophoresis or inefficiently formed upon CINV loading. At the same time, efficient siMDR1 loading was observed in control experiments with LF (Supplementary Figure S8C) and 2X3-DOPE liposomes (Supplementary Figure S8D), manifested by a step-by-step disappearance of the band corresponding to siMDR1. Taking into account that similar results were obtained in the electrophoretic analysis of µ-ON-21/B16 CINV complexes, we hypothesized that i) this approach was not applicable for the analysis of complexes of CINVs with short tNAs and ii) short RNA duplexes might not be loaded into CINVs/aCINVs.

### 3.4 Delivery of isRNA by CINVs

**FIGURE 5:**
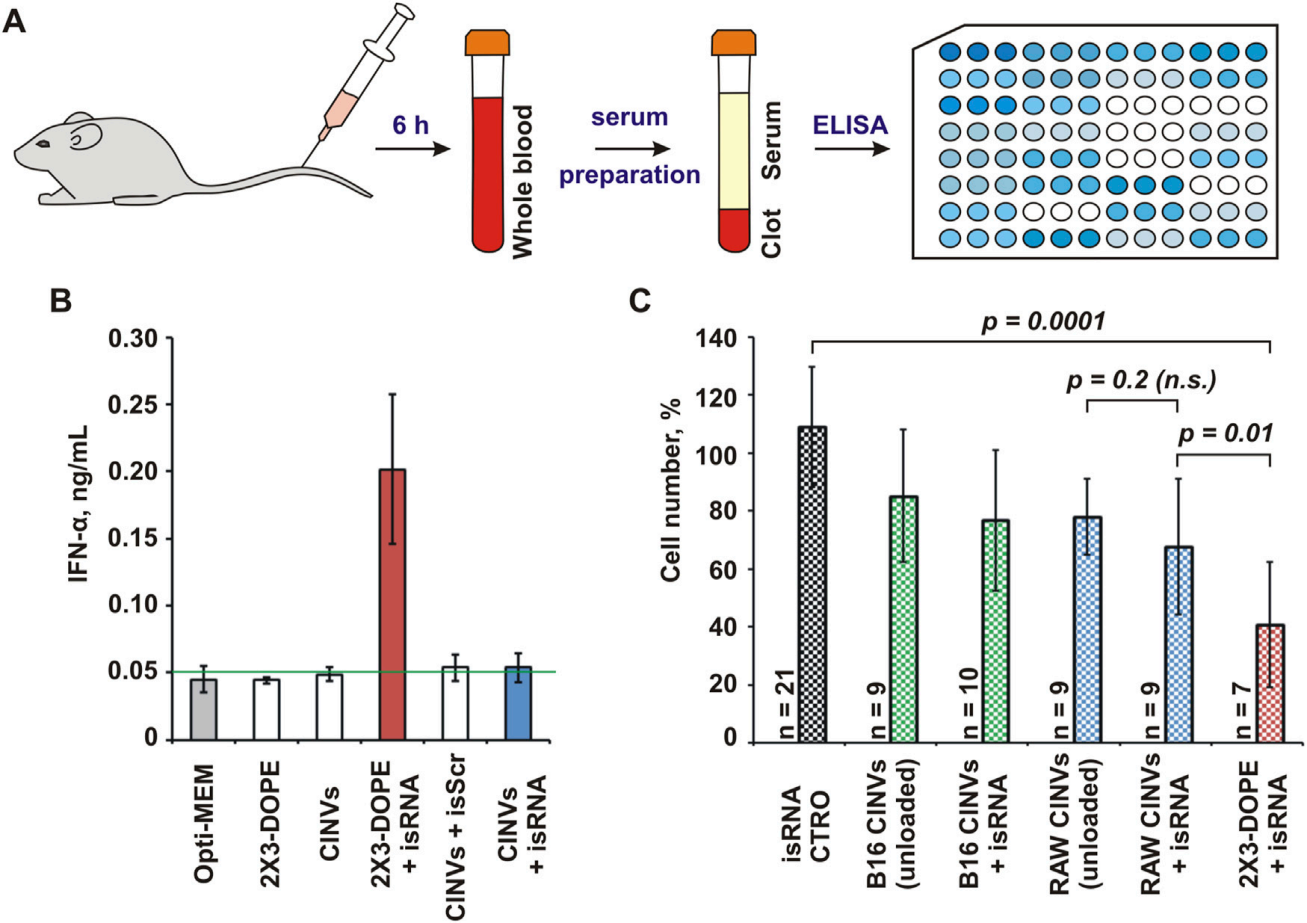
Biological activity of isRNA loaded into CINVs. **(A)** Intravenous administration of RAW CINVs loaded with isRNA into CBA mice; scheme of experiments. **(B)** The IFN-α concentration in serum samples of mice treated with isRNA loaded/pre-complexed with either RAW CINVs or 2X3-DOPE liposomes. Measurements were performed in duplicate. The blank (0.05 ng/mL) was indicated by a green line. **(C)** Anti-proliferative activity of isRNA in B16 cells after B16 CINV-, RAW CINV-, or 2X3-DOPE-mediated delivery; WST-1 assay. The absorbance measured in the control cells was set at 100%. Self-penetration of isRNA in B16 cells is indicated by CTRO. The number of measurements is indicated by n. Data are presented as mean and SD; *n.s.*–not significant.

Another class of short RNA duplexes used for loading into CINVs was immunostimulatory RNA (Kabilova et al., 2012) (Table 1; isRNA), which increased the level of IFN-α in the serum of immunocompetent mice 6 h after i.v. administration in complexes with 2X3-DOPE liposomes (Kabilova et al., 2017). The biological activity of isRNA loaded into RAW CINVs was assessed in CBA mice by analyzing IFN-α level in the blood serum using ELISA (Figure 5A). We observed that similar to siMDR1 delivery, CINVs were unable to functionally deliver isRNA (RAW CINVs in this case) since the level of IFN-α remained unaltered after isRNA/CINV administration (Figure 5B).

Along with increasing the secretion of some cytokines, isRNA exhibits an anti-proliferative effect on tumor cells *in vitro* (Bishani and Chernolovskaya, 2021). In particular, isRNA can slow down the division of B16 cells after 2X3-DOPE-mediated delivery (Bishani et al., 2023). Therefore, we examined the functional delivery of isRNA loaded into B16 CINVs or RAW CINVs by assessing cell proliferation using the WST-1 assay (Figure 5C). To detect the biological effect in these experiments, the amount of CINVs relative to cells was increased from 20 μg to 100 µg per well. The data displayed in Figure 5C showed that the anti-proliferative effect of unloaded CINVs used at high concentrations on B16 cells was similar to that we observed previously in other cells (Oshchepkova et al., 2021). However, isRNA loaded into CINVs did not cause as strong an anti-proliferative effect in B16 cells as caused by isRNA/2X3-DOPE lipoplexes; however, a tendency to slow down the proliferation of B16 cells was detected for isRNA/RAW CINVs (Figure 5C). Since the differences between unloaded and isRNA-loaded CINVs were statistically insignificant, this again indicates a lack of functional delivery of isRNA. Thus, based on the absence of biological effects of both siRNA and isRNA, we can conclude that short RNA duplexes cannot be loaded into CINVs/aCINVs using the methods applied.

### 3.5 Delivery of pDNA by CINVs

**FIGURE 6:**
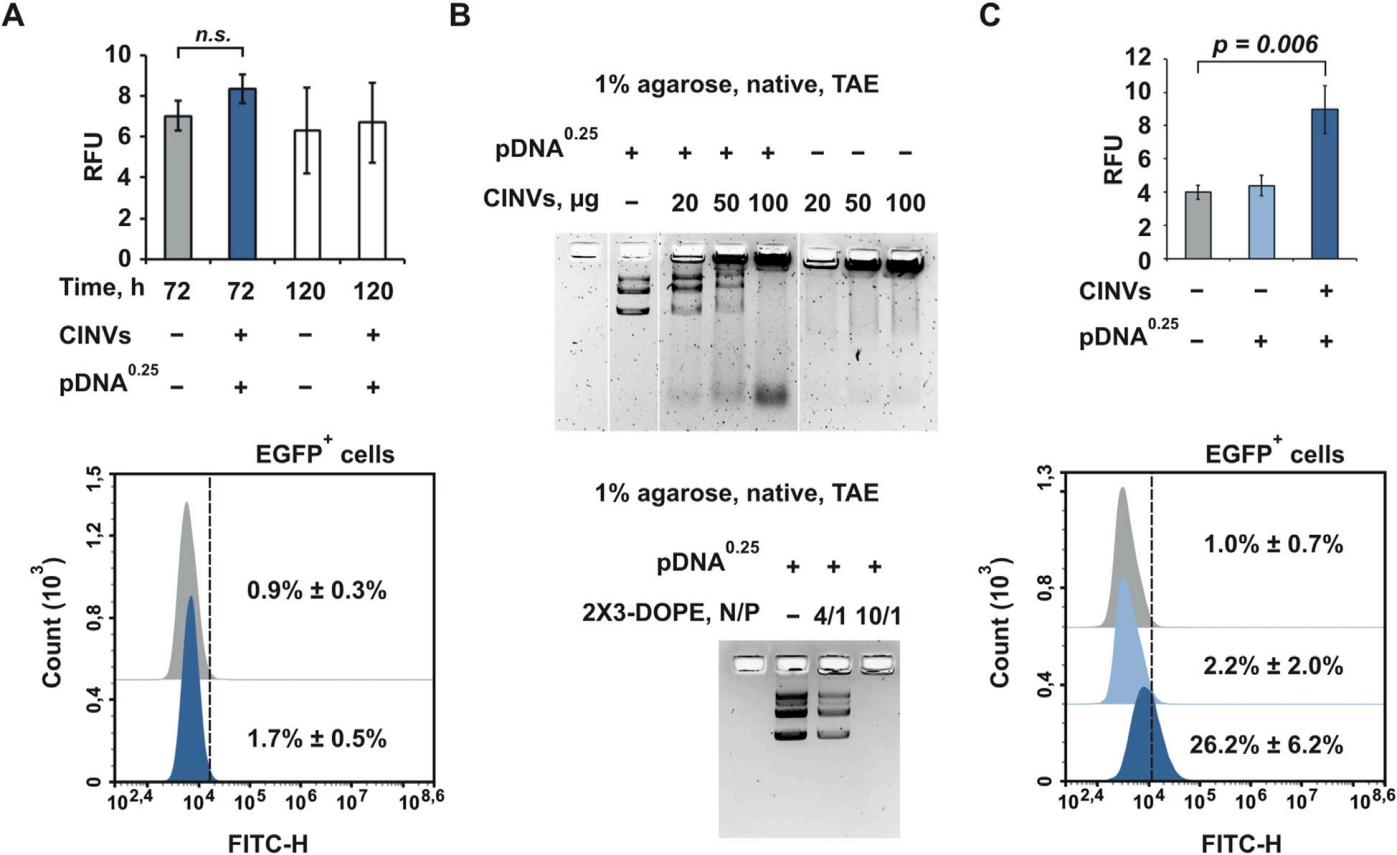
CINV-mediated delivery of pDNA. **(A)** Delivery of pDNA by KB CINVs (100 µg/well) to KB-3-1 cells; the experimental time was 72–120 h (n = 3–4). **(B)** Gel shift assay of pDNA loaded into KB CINVs (n = 2); the complexes of pDNA with 2X3-DOPE liposomes were used as positive controls (n = 2). **(C)** Delivery of pDNA by B16 CINVs (100 µg/well) to HEK 293 cells; the experimental time was 72 h (n = 4–6). **(A, C)** Flow cytometry assay data. The amount of pDNA (µg) in the loading mixture is indicated in superscript. Data are presented as mean and SD; *n.s.*–not significant.

The CINV-mediated delivery of pDNA was studied using the pEGFP-C2 vector encoding the green fluorescent protein EGFP. First, we analyzed the delivery of pDNA (0.25 or 1.5 µg) to KB-3-1 cells using 20 (Supplementary Figure S9) or 100 (Figure 6A) μg KB CINVs. Because we did not detect EGFP expression regardless of the experimental conditions (including changing the amount of CINVs or pDNA and using the incubation time of 72–120 h), we incubated KB-3-1 cells with 50 µM chloroquine for 24 h to improve the possible low endosomal escape of pDNA/KB CINV complexes (Supplementary Figure S10A). However, cell treatment with chloroquine did not improve CINV-mediated pDNA delivery in KB-3-1 cells (Supplementary Figure S10B), contrary to the significant stimulation of EGFP expression after 2X3-DOPE-mediated pDNA delivery (Supplementary Figure S10C). In addition, the inefficient loading/binding of pDNA with CINVs could lead to the absence of EGFP expression in recipient cells. Surprisingly, the pDNA/KB CINV complexes were detected by gel shift analysis (Figure 6B), indicating an efficient loading process mediated by the Fr-Th procedure: a dose-dependent effect of CINVs on the disappearance of bands corresponding to pDNA was observed.

We previously observed that the efficiency of CINV uptake by cells was mainly determined by the type of recipient cells and was less dependent on the source of the vesicles (Oshchepkova et al., 2021). The detection of the pDNA/KB CINV complexes in the agarose gel suggests that KB-3-1 cells may not be suitable for CINV-mediated pDNA delivery for unknown reasons. The use of other cell lines may offer prospects for detecting CINV-mediated pDNA delivery. Mouse melanoma B16 cells (Supplementary Figure S11) and HEK 293 cells (Figure 6C) were chosen for these experiments. It was observed that both B16 CINVs and RAW CINVs exhibited poor pDNA delivery in B16 cells: less than 5% of cells were EGFP positive with low RFU, and increasing the amount of CINVs from 100 to 200 µg did not result in a dose-dependent improvement (Supplementary Figure S11). It should be noted that 2X3-DOPE-mediated pDNA delivery in B16 cells was also ineffective.

In contrast to KB-3-1 or B16 cells, the delivery of pDNA to HEK 293 cells by B16 CINVs was functional and resulted in sufficiently strong EGFP expression: on average, 26% of the cells were EGFP-positive, and RFU increased twofold compared with control cells (Figure 6C). Thus, CINVs in the amount of at least 100 μg per well can form complexes with pDNA (0.25 μg) and ensure its functional delivery into recipient cells. Moreover, the efficiency of delivery depends on the type of recipient cells.

## 4 Discussion

In this study, we examined the ability of CINVs/aCINVs to deliver tNAs differing in length, modification patterns, and structure into a range of cells and assessed the efficiency of delivery by measuring their biological activity. We observed efficient functional delivery of µ-ON-21 resulting in a twofold reduction in the level of miR-21 and efficient functional delivery of pDNA. In the latter case, our findings indicated that the efficiency of pDNA delivery depended on the type of recipient cells and the concentration of loaded CINVs in the medium; the most efficient delivery was observed in HEK 293 cells when 100 µg/well of pDNA-loaded CINVs were used (Figure 6C). Under similar conditions, no CINV-mediated pDNA delivery was observed in KB-3-1 and B16 cells. These results are consistent with our previous observation that the efficiency of CINV uptake by cells depends on the type of recipient cells (Oshchepkova et al., 2021). The successful delivery of µ-ON-21 lets us suggest that it may localize within both the cytoplasm and nucleus of cells after CINV-mediated delivery, as ASO activity can occur in both areas (Gagliardi and Ashizawa, 2021). Nonetheless, examination of the intracellular localization of B16 CINVs and FAM-µ-ON-21/B16 CINVs by confocal microscopy revealed their predominant accumulation in the cytoplasm (Figures 2F, 3; Supplementary Figure S2A).

Surprisingly, CINVs/aCINVs did not provide the functional delivery of short RNA duplexes (siRNA and isRNA) under any experimental conditions. Being chemically different, siRNA is a 2′-F, 2′-OMe fully modified RNA, while isRNA has a natural ribose-phosphate backbone. Both molecules are 21–22 base-pair RNA duplexes with two (siRNA) or three (isRNA) 3′-overhangs. A thorough analysis showed that the following factors could be the reasons for the ineffective CINV-mediated delivery of siRNA and isRNA in our experiments: i) the RNA duplexes became entrapped in endosomes or unproductively accumulated inside cells; ii) the complexes of CINVs/aCINVs with siRNA or isRNA either did not form or were rapidly destroyed in the environment.

To improve the low endosomal escape of tNAs, cells can be incubated with various chemical agents, including sucrose (Ciftci and Levy, 2001; Caron et al., 2004), which acts as a lysosomal/endosomal swelling agent. Since we did not observe a positive effect of sucrose treatment on siRNA functional delivery (Supplementary Figure S4C), we speculate that siRNA is not delivered into the cells by CINVs. Further examination of the complexes of siRNA/CINVs using the gel shift assay revealed that they either did not form or were unstable during gel electrophoresis (Supplementary Figure S8A, B). Thus, we hypothesize that the inefficient procedure of siRNA loading into CINVs/aCINVs or the instability of siRNA/CINV complexes under experimental conditions are responsible for the failure of siRNA delivery. The use of different loading strategies or recipient cells (K562-MDR1-GFP instead of KB-3-1-MDR1-GFP cells) and variations of siRNA or CINV/aCINV concentrations in the loading mixture did not lead to the appearance of silencing activity of the delivered siRNA (Figures 4A, B; Supplementary Figures S4A, S4B, S5A, S7C).

The functional CINV-mediated delivery of isRNA, another short RNA duplex, was studied *in vivo* and *in vitro*. To achieve immunostimulation, isRNA/CINV complexes must be taken up by blood phagocytes. The ability of CINVs to be internalized by monocytes was previously shown in the culture of peripheral blood mononuclear cells (Gomzikova et al., 2020). Nonetheless, we did not observe the elevation of IFN-α levels following i.v. administration of isRNA/RAW CINV complexes in CBA mice (Figure 5B). Apparently, isRNA was either not loaded into CINVs or the resulting complexes were not sufficiently stable in the bloodstream. Similar results were obtained *in vitro* (Figure 5C). Moreover, under the conditions where effective CINV-mediated delivery of µ-ON-21 was observed (Figure 2A), specifically B16 CINVs and B16 cells, no anti-proliferative effect of isRNA was found (Figure 5C).

Based on our previous results (Oshchepkova et al., 2019; Oshchepkova et al., 2021), we chose the Fr-Th method for loading tNAs into CINVs/aCINVs. The problem of short RNA duplex loading into CINVs/aCINVs can probably be addressed by changing the loading strategy. Pre-delivery of therapeutics to vesicle-producing cells can be an alternative to the Fr-Th method used in this study. Unlike natural EVs, CINVs/aCINVs are capable of randomly incorporating the internal content of their parent cell. For this reason, pre-delivery has been adapted for packaging several proteins into CINVs through lentiviral transduction of cells (Chulpanova et al., 2021a; Chulpanova et al., 2021b; Shkair et al., 2022; Alatrash et al., 2023; Chulpanova et al., 2023; Filin et al., 2023a; Filin et al., 2023b). The advantage of this strategy is the internal localization of the cargo of interest in CINVs/aCINVs. At the same time, there is always debate about the location of therapeutics after loading into previously isolated vesicles. For example, the Khvorova’ group has shown that the majority of hydrophobically modified siRNAs are located on the surface but not inside EVs after loading (Didiot et al., 2016; Biscans et al., 2018). It should be noted that most tNAs require chemical modification patterns for successful clinical application; therefore, they must be delivered to vesicle-producing cells in a ready-to-use form and cannot be overexpressed inside them.

Given the high cost of producing chemically modified tNAs, the choice of loading strategy also depends on the therapeutic doses of CINVs/aCINVs. According to our previous observations, approximately 100–400 million cells are needed to produce 1 mg of CINVs/aCINVs (total protein) (Oshchepkova et al., 2021). The doses of CINVs varied depending on the experimental design, route of administration, and choice of experimental animals. For example, mice received 10 µg (Zhdanova et al., 2022) or 15 µg (Shkair et al., 2022) CINVs for intranasal or subcutaneous administration, respectively; rats received up to 50 μg CINVs during i.v. injection (Kostennikov et al., 2022). Here, we used 200 μg CINVs per mouse for i.v. injection (see Section 3.4), which was similar to the dosage (300 μg CINVs) previously used for intrathecal administration to a pig (Shulman et al., 2023). Therefore, the choice of CINV loading strategy is determined by the cost/effectiveness ratio and requires direct comparison and additional study.

Cell treatment with chemical agents is one of the simplest and most practical ways to produce EV mimetics or increase the production of natural EVs. However, two factors should be considered when using this approach. First, the composition of EV mimetics may be altered by the chemicals used. However, this risk can be minimized by shortening cell treatment with chemical agents. In particular, the preparation of CINVs/aCINVs includes a 30-min incubation with Cyt B, which is unlikely to affect the contents of the vesicles and the cells producing them. Therefore, the CINV/aCINV preparation method appears promising in terms of safety and the time required for vesicle isolation.

The second point to consider is the safety issue related to the possible presence of chemicals in the EV mimetic preparations. Previously, we quantified Cyt B in CINVs using mass spectrometry analysis (Oshchepkova et al., 2021) and hypothesized that even after extending the washing step to three rounds (Figure 1A), CINVs/aCINVs might still contain a residual amount of Cyt B. The presence of Сyt B in CINVs/aCINVs was supported by the observation that B16 CINVs had the opposite dose-dependent effect on B16 cell division: the vesicles stimulated cell division at a low concentration (Supplementary Figure S1A) and delayed it at a high dose (Figure 5C). This is reminiscent of the impact of free Cyt B on the proliferation of L929 cells (Oshchepkova et al., 2021). We also speculate that another effect of CINVs on recipient cells may be explained by the presence of Cyt B in the vesicle preparations. Specifically, when mesenchymal stem cells (MSCs) were incubated with CINVs, the stem cell markers of these cells decreased, mimicking the beginning of the differentiation process (Solovyeva et al., 2022). In addition, Cyt B itself may imitate the process of cell differentiation in some cases (Bianconi et al., 2022) and can enhance the adipogenic or osteogenic potential of MSCs in a corresponding induction medium (Bianconi et al., 2022; Pampanella et al., 2023).

It can be assumed that the presence of Cyt B traces in CINV/aCINV preparations may be useful for vesicle application in anticancer therapy because some chemotherapy drugs may exhibit enhanced therapeutic effects after loading into vesicles compared with their use in a free form. For instance, doxorubicin loaded into CINVs demonstrated increased toxicity to ACHN cells compared with conventional chemotherapy (Nair et al., 2021). Similarly, methotrexate packaged into EVs killed H22 cells almost 12-fold more effectively than the equivalent amount of free drug (Tang et al., 2012).

Summarizing, we conclude that CINVs/aCINVs represent a promising tool for ASO-based therapy, with limited utility for other types of tNAs. It should also be mentioned that the *in vitro* delivery conditions used in our experiments are characterized by a reduced influence of non-cellular blood components due to the use of the EV-depleted FBS. Further animal experiments using different routes of CINV administration will help to elucidate their potential in the *in vivo* system. The complex biological organization of CINVs/aCINVs, including a variety of surface molecules that can promote their tissue-specific accumulation and internal components inherited from parental cells that can exert their own therapeutic effects, is a significant advantage over synthetic carriers. Moreover, we hypothesize that the chemical component of CINVs/aCINVs may be beneficial and enhance the overall biological effect on recipient cells. However, given the limitations in the use of CINVs for the delivery of RNA duplexes, the search for new chemical compounds to create EV mimetics or the optimization of approaches for loading tNAs into CINVs may be relevant in the future. Furthermore, it is crucial to acknowledge that CINVs, similar to other cell-based delivery systems, do not address the fundamental challenge of finding the most suitable cell source for clinical application. This highlights the need for continued research and development to ensure the optimal use of CINVs in clinical conditions.

## Data Availability

The original contributions presented in the study are included in the article/Supplementary Material, further inquiries can be directed to the corresponding author.
