# Supplementary material for: Extracellular vesicle mimetics as delivery vehicles for oligonucleotide-based therapeutics and plasmid DNA
Source: Front Bioeng Biotechnol. 2024 Oct 17;12:1437817. doi: 10.3389/fbioe.2024.1437817 (PMC11528538; doi:10.3389/fbioe.2024.1437817)
Supplement: Supplementary file 1 [file DataSheet1.pdf]

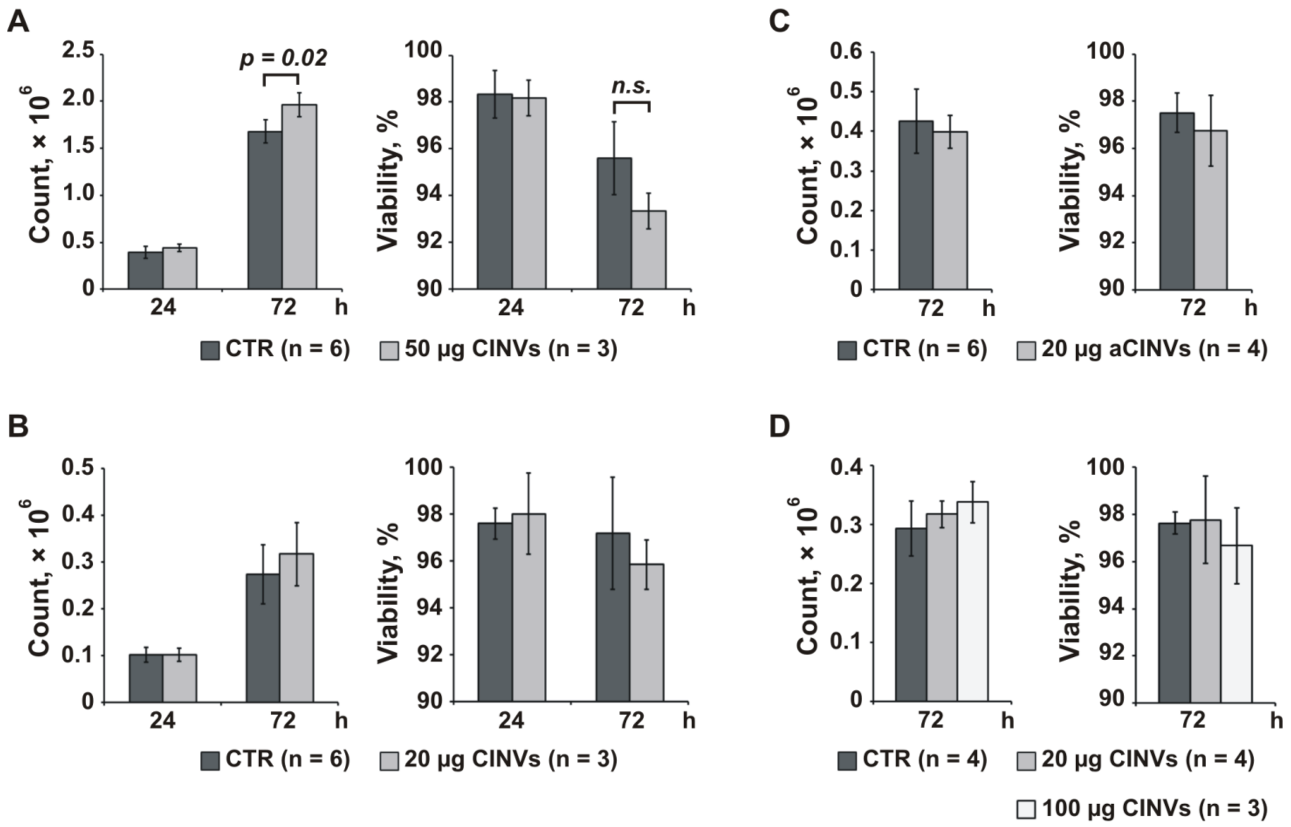

Supplementary Figure S1. Trypan blue assay of cell count and viability after incubation with unloaded CINVs/aCINVs: B16 **(A)**, KB-3-1-MDR1-GFP **(B)**, K562-MDR1-GFP **(C)**, and KB-3-1 cells **(D)** were incubated in the presence of B16 CINVs (50 µg/well), KB CINVs (20 µg/well), K562 aCINVs (20 µg/well), and KB CINVs (20–100 µg/well), respectively. The number of measurements is indicated by n. Data are presented as mean and standard deviation (SD). KB–KB-3-1; RAW–Raw 264.7; aCINVs–vesicles prepared from late apoptotic/necrotic cells; *n.s.*–not significant.

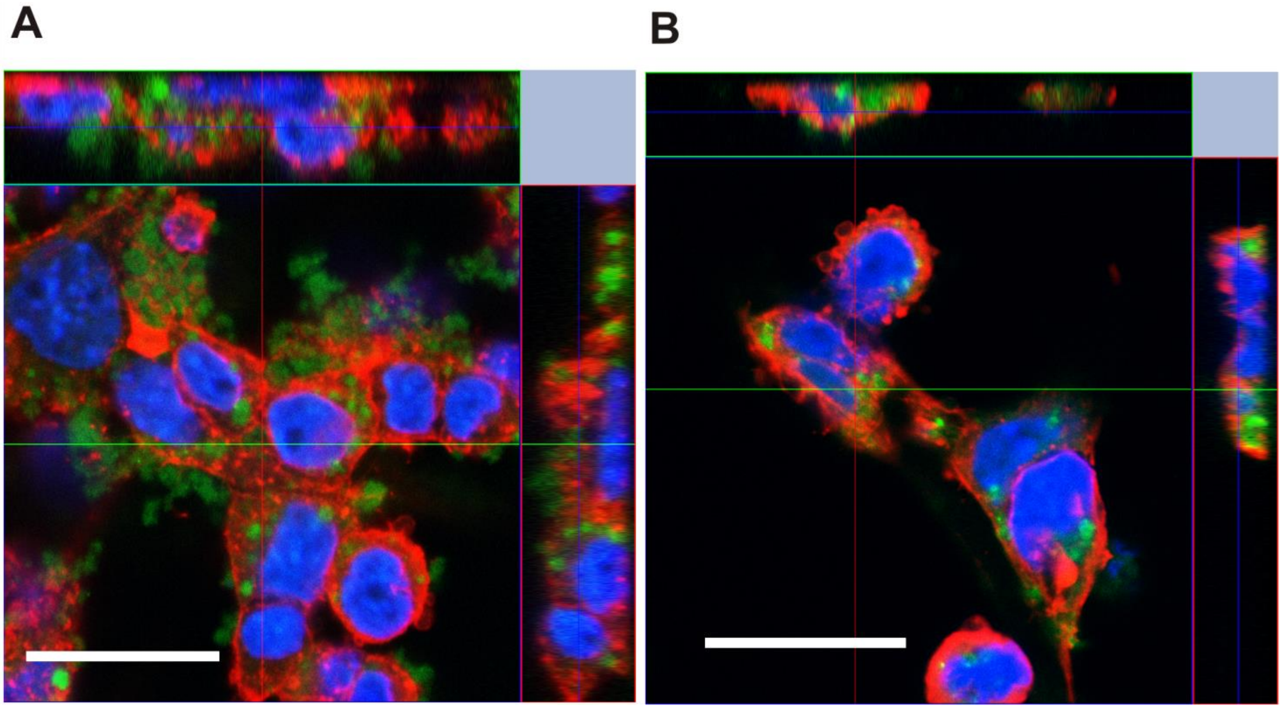

Supplementary Figure S2. Confocal microscopy analysis (Z-stack images) of B16 cells incubated with either unloaded B16 CINVs stained with CFSE dye **(A)** or 2X3-DOPE liposomes pre-complexed with FAM-labeled μ-ON-21 (FAM-μ-ON-21) **(B)**. The nuclei are indicated by blue color, actin filaments–by red color, FAM-μ-ON-21 or CFSE is indicated by green signal. The scale bar is 20 μm.

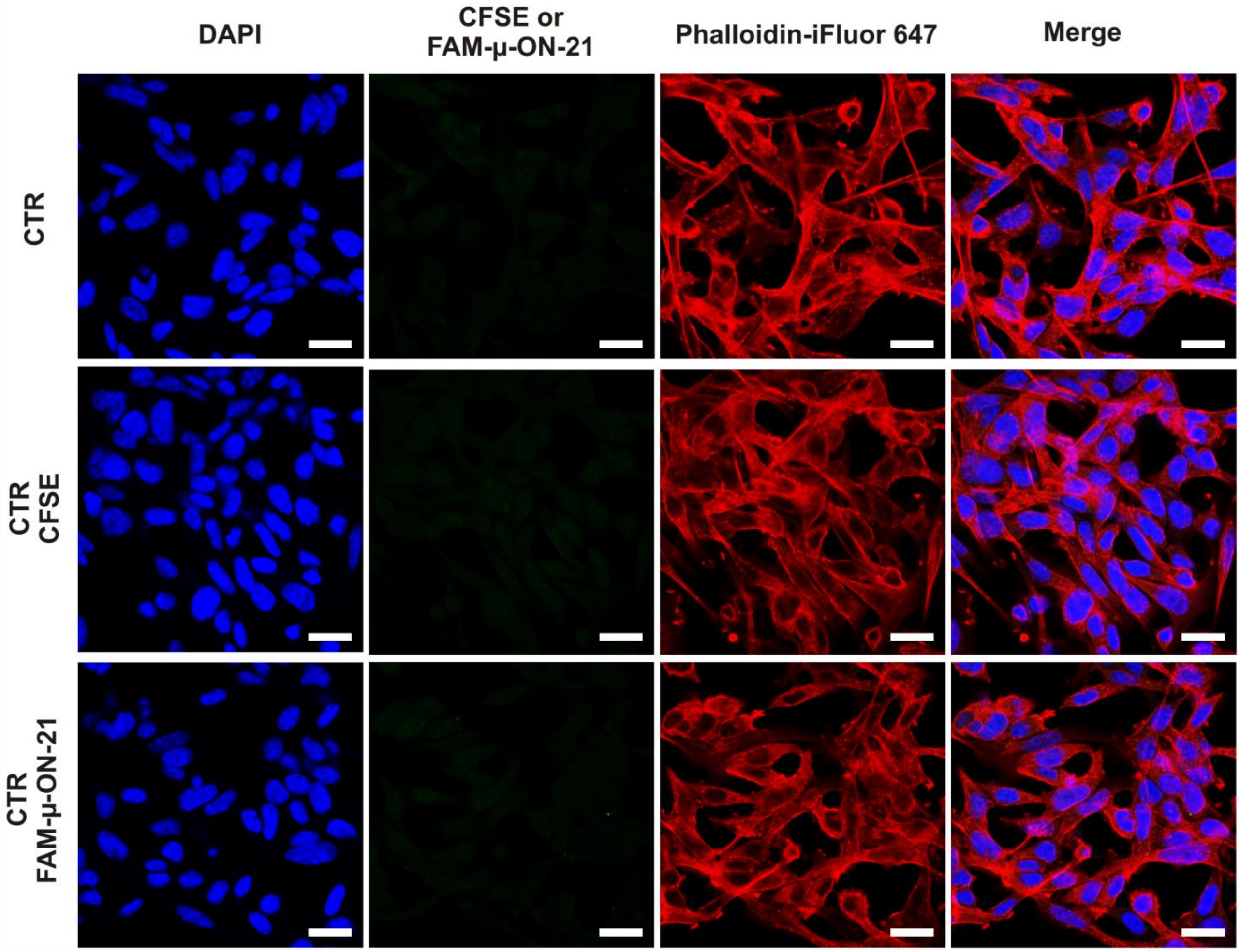

Supplementary Figure S3. Confocal microscopy analysis of B16 cells incubated with control samples. CTR–control untreated cells. CTR CFSE–nonspecific cell staining with CFSE dye: B16 cells were incubated with CFSE dye, which was subjected to all procedures of CINV staining but without vesicles. CTR FAM-μ-ON-21 represents the control of oligonucleotide self-penetration into the cells. The nuclei are indicated by blue color, actin filaments–by red color, CFSE or FAM-μ-ON-21 is indicated by green signal. All images in the "green" channel were made with identical settings. The scale bar is 20 μm.

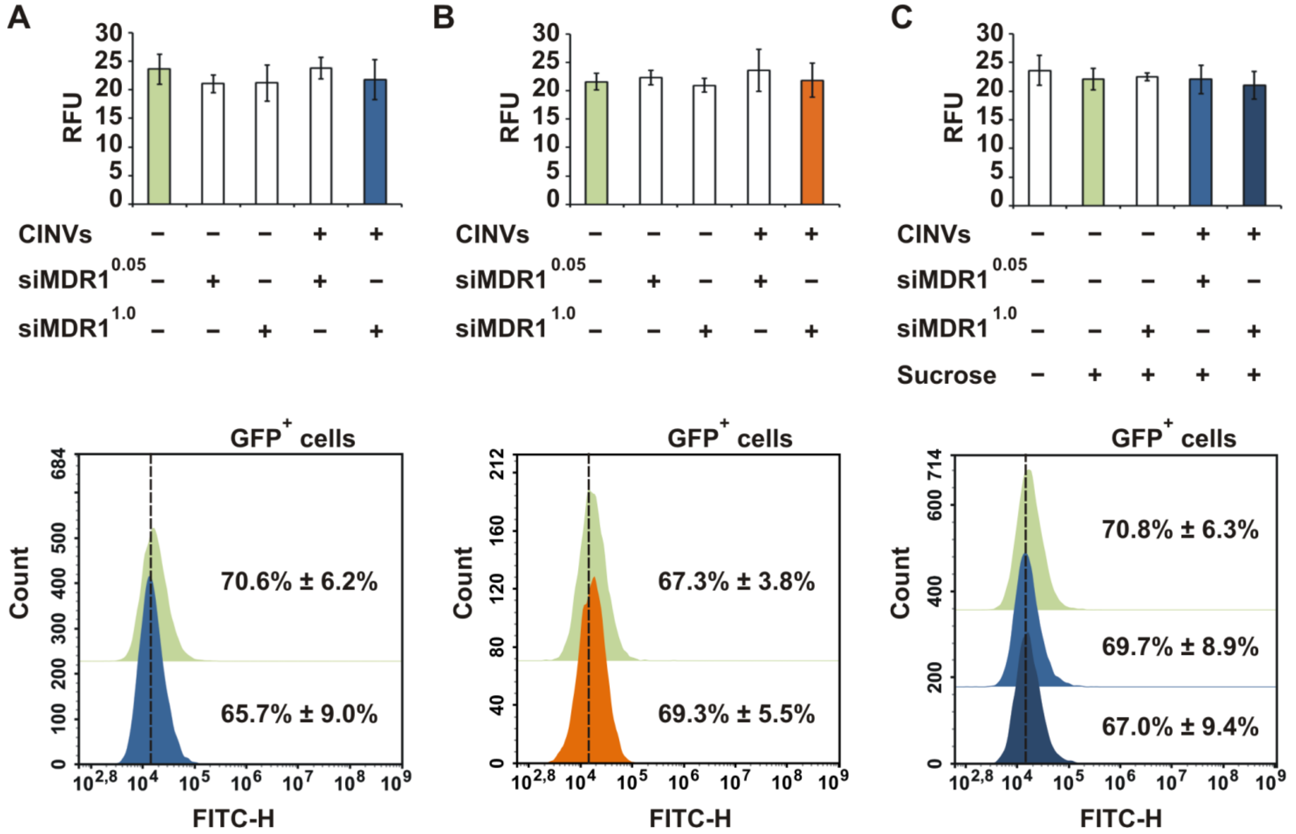

Supplementary Figure S4. Delivery of siMDR1 by KB CINVs to KB-3-1-MDR1-GFP cells. Flow cytometry assay data (n = 3-8). KB CINVs (20 µg/well) were loaded with siMDR1 either by Fr–Th **(A, C)** or by chemical permeabilization **(B)**. **(C)** Delivery of siMDR1 by KB CINVs in the presence of 0.2 M sucrose; cell viability under these conditions did not decrease below 98% according to the trypan blue assay. Summary fluorescence data are represented as relative fluorescence units (top graphs; RFU) and percentage of GFP-positive cells (bottom histograms; count vs. FITC-H). The superscript indicates the amount (nmol) of siMDR1 used in the loading mixture. Data are presented as mean and SD.

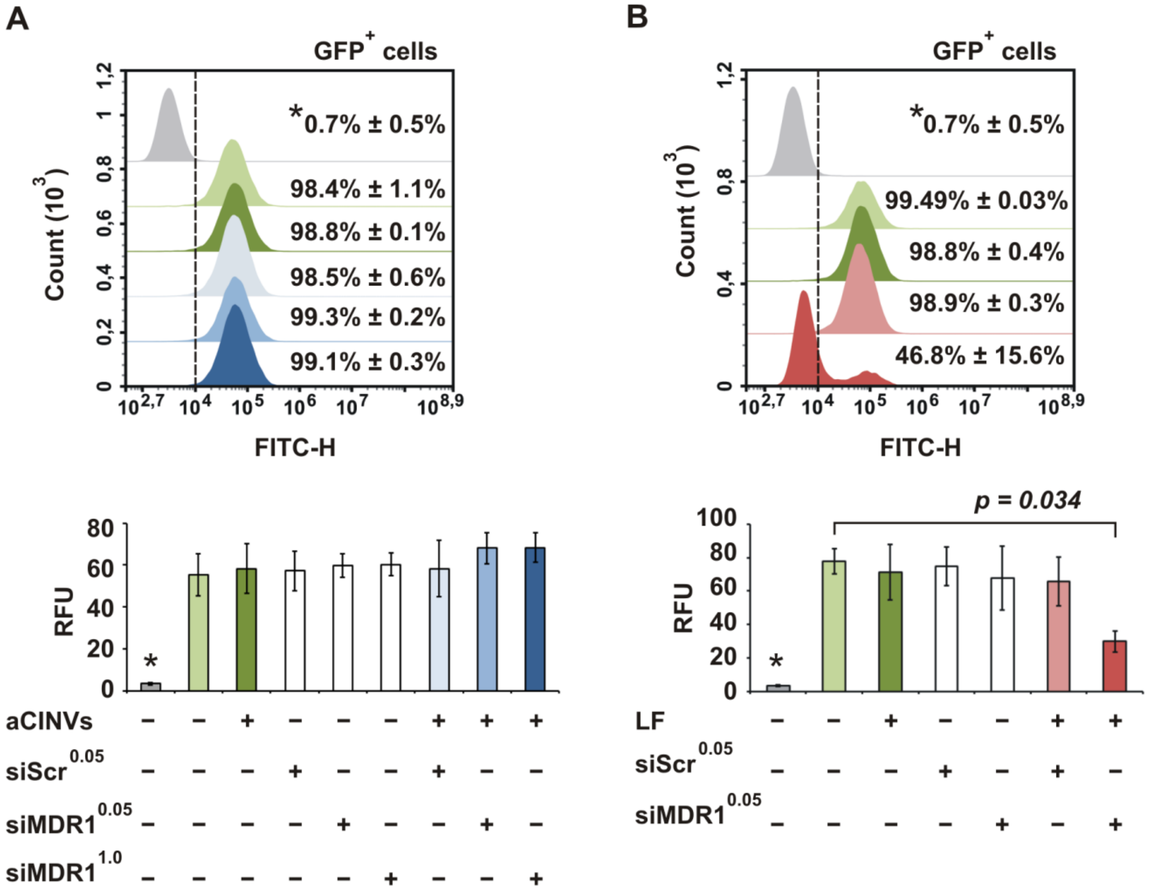

Supplementary Figure S5. Delivery of siMDR1 to K562-MDR1-GFP cells by K562 aCINVs **(A)** or Lipofectamine 2000 (LF) **(B)**. Flow cytometry assay data. K562 aCINVs were generated from late apoptotic/necrotic K562 cells based on earlier observations indicating that K562 cells exhibited increased internalization of K562 aCINVs compared with vesicles prepared from live cells (Oshchepkova et al., 2021). The superscript indicates the amount (nmol) of siMDR1 or siScr (scrambled) used in the loading mixture. Parental K562 cells that do not express GFP are indicated by an asterisk (*). The number of measurements of K562 cells (*) was 7; in other groups, it varied from 3 to 4. Data are presented as mean and SD.

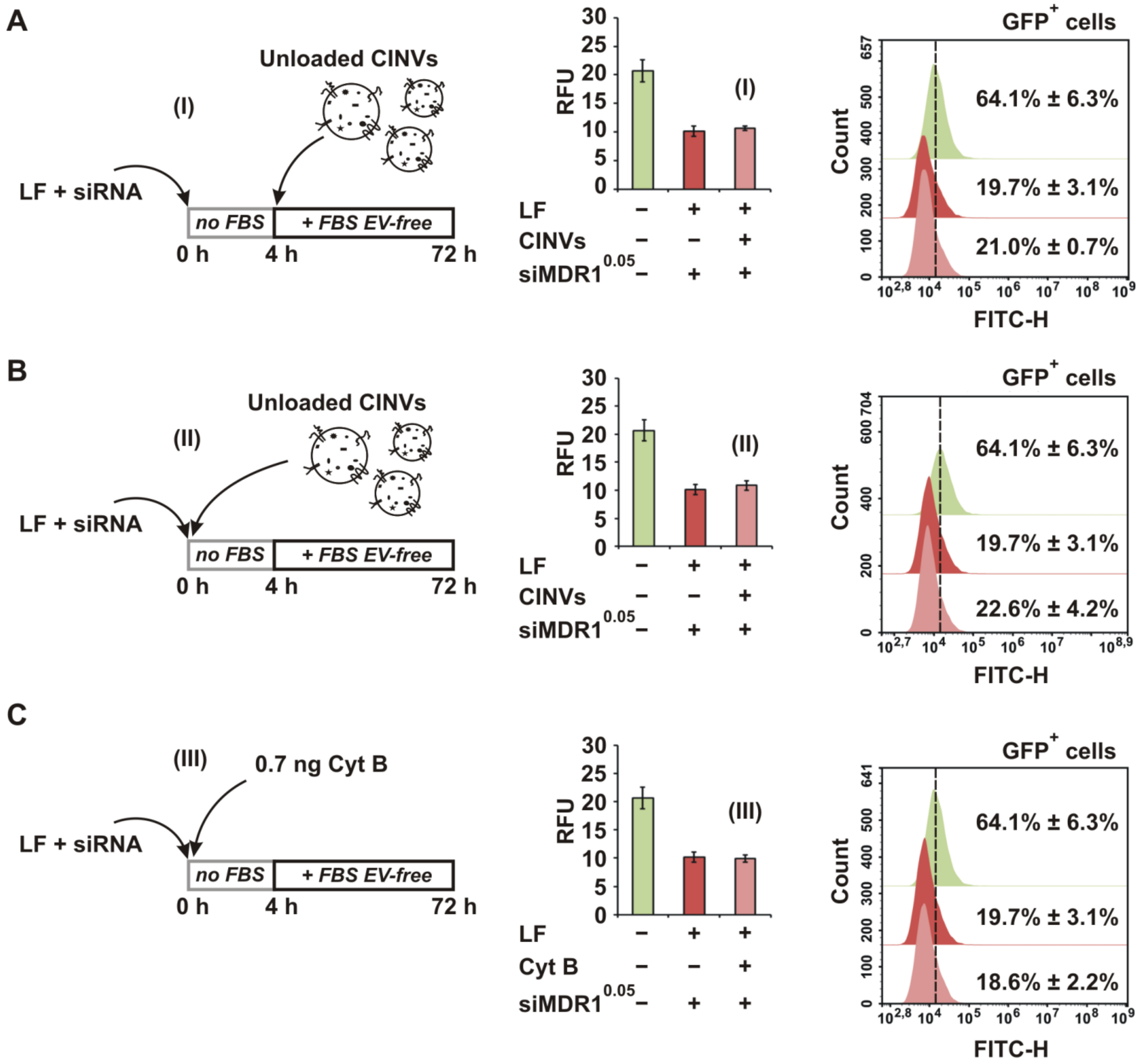

Supplementary Figure S6. Effects of KB CINVs or cytochalasin B (Cyt B) on the LF-mediated delivery of siMDR1 in KB-3-1-MDR1-GFP cells. Flow cytometry assay data (n = 4-7). **(A)** Unloaded KB CINVs (20 µg/well) were added to cells after lipofection. **(B)** Unloaded KB CINVs (20 µg/well) were added simultaneously with lipofection. **(C)** According to our previous study, 20 µg CINVs (total protein) contained ~ 0.7 ng Cyt B (Oshchepkova et al., 2021); therefore, 0.7 ng Cyt B was added to cells simultaneously with lipofection. The superscript indicates the amount (nmol) of siMDR1 used in the loading mixture. Data are presented as mean and SD.

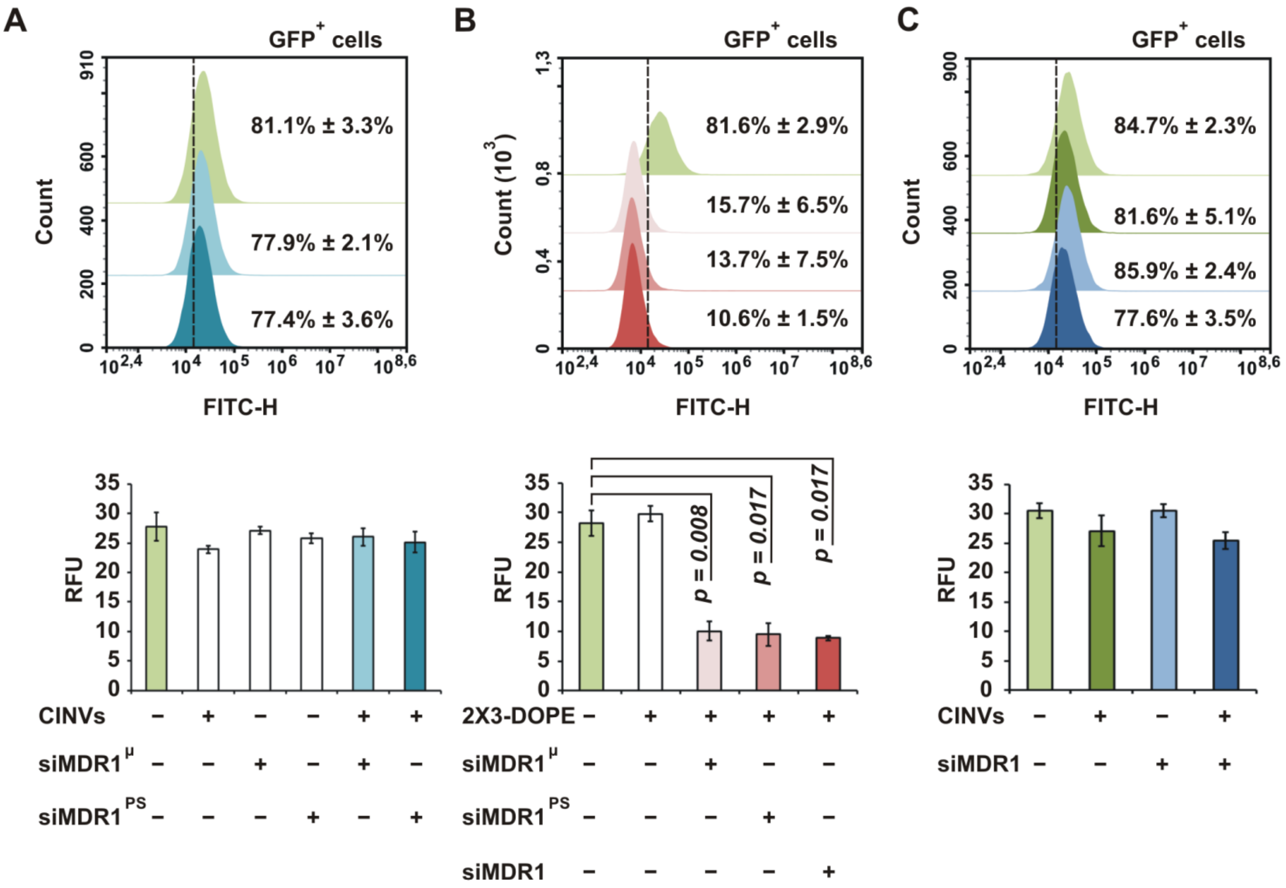

Supplementary Figure S7. Delivery of siMDR1 containing phosphorothioate (siMDR1^{PS}) or mesyl (siMDR1$^{\mu}$) modifications to KB-3-1-MDR1-GFP cells by KB CINVs (20 µg/well) **(A)** or 2X3-DOPE liposomes **(B)**. **(C)** Delivery of siMDR1 by 100 µg/well KB CINVs to KB-3-1-MDR1-GFP cells. The amount of siRNA in the loading mixture was 0.05 nmol. Flow cytometry assay data (n = 3-7). Data are presented as mean and SD.

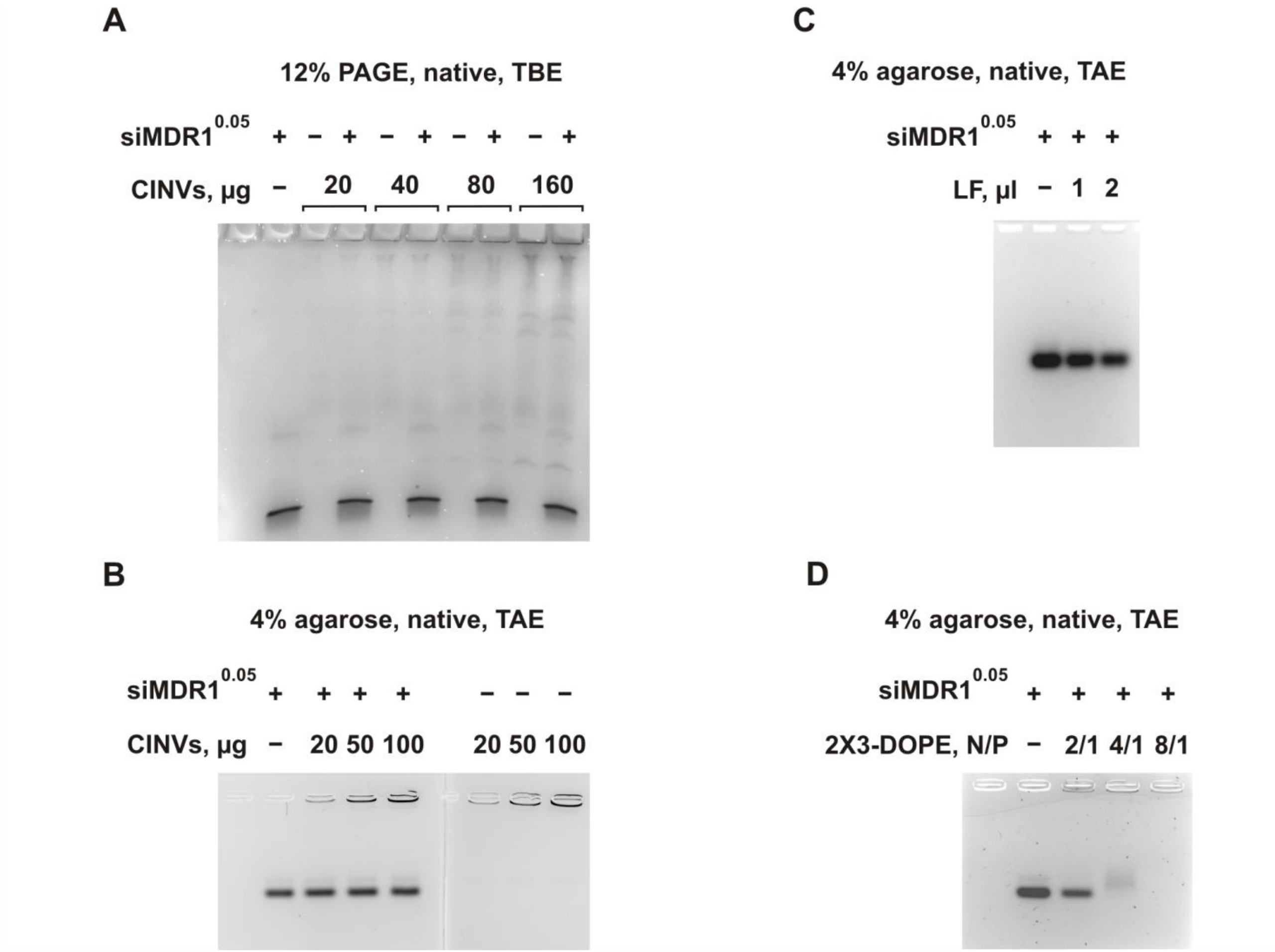

Supplementary Figure S8. Gel shift assay of siMDR1 loaded into KB CINVs (n = 2-3) **(A-B)**. Complexes of siMDR1 with LF **(C)** or 2X3-DOPE liposomes **(D)** were used as positive controls (n = 2-3). The superscript indicates the amount (nmol) of siMDR1 in the loading mixture. Data are presented as mean and SD.

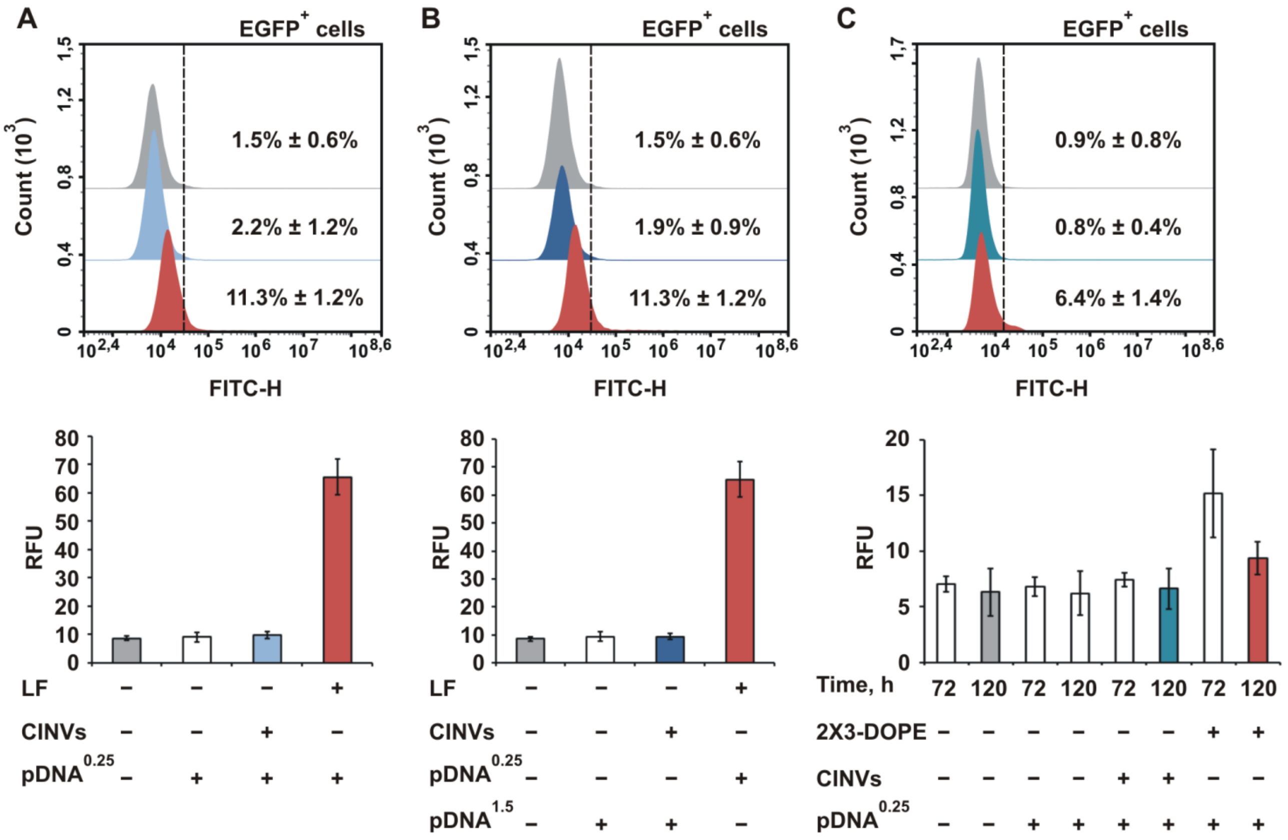

Supplementary Figure S9. Delivery of pDNA by KB CINVs (20 µg/well) to KB-3-1 cells. Flow cytometry assay data (n = 3-6). The amount of pDNA (0.25 or 1.5 µg) in the loading mixture is indicated in superscript. The experimental time was 72 h **(A, B)** or 72–120 h **(C)**. Data are presented as mean and SD.

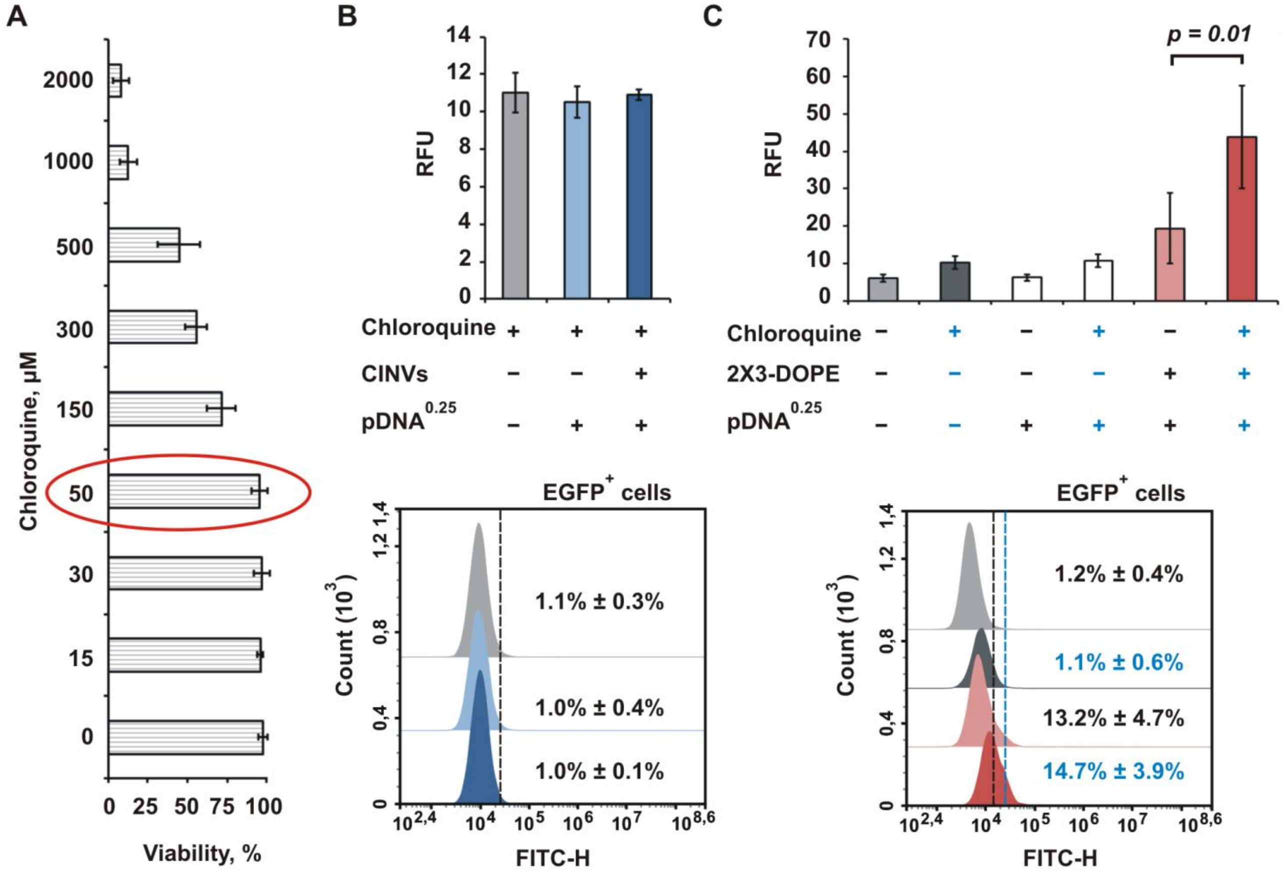

Supplementary Figure S10. Delivery of pDNA to KB-3-1 cells in the presence of 50 μM chloroquine. **(A)** Viability of KB-3-1 cells (trypan blue assay) after 24-h incubation with different concentrations of chloroquine (n = 3-7). **(B)** Delivery of pDNA by KB CINVs (100 μg/well) in the presence of 50 μM chloroquine. **(C)** Expression of pDNA in KB-3-1 cells after 2X3-DOPE-mediated delivery in the presence of 50 μM chloroquine. **(B-C)** Flow cytometry assay data (n = 3-6). Chloroquine exposure during pDNA delivery by KB CINVs **(B)** or 2X3-DOPE **(C)** lasted for 24 h after pre-incubation of cells with the complexes for 4 h under chloroquine-free conditions; the total experimental time was 72 h. The superscript indicates the amount of pDNA (μg) used in the loading mixture. Data are presented as mean and SD.

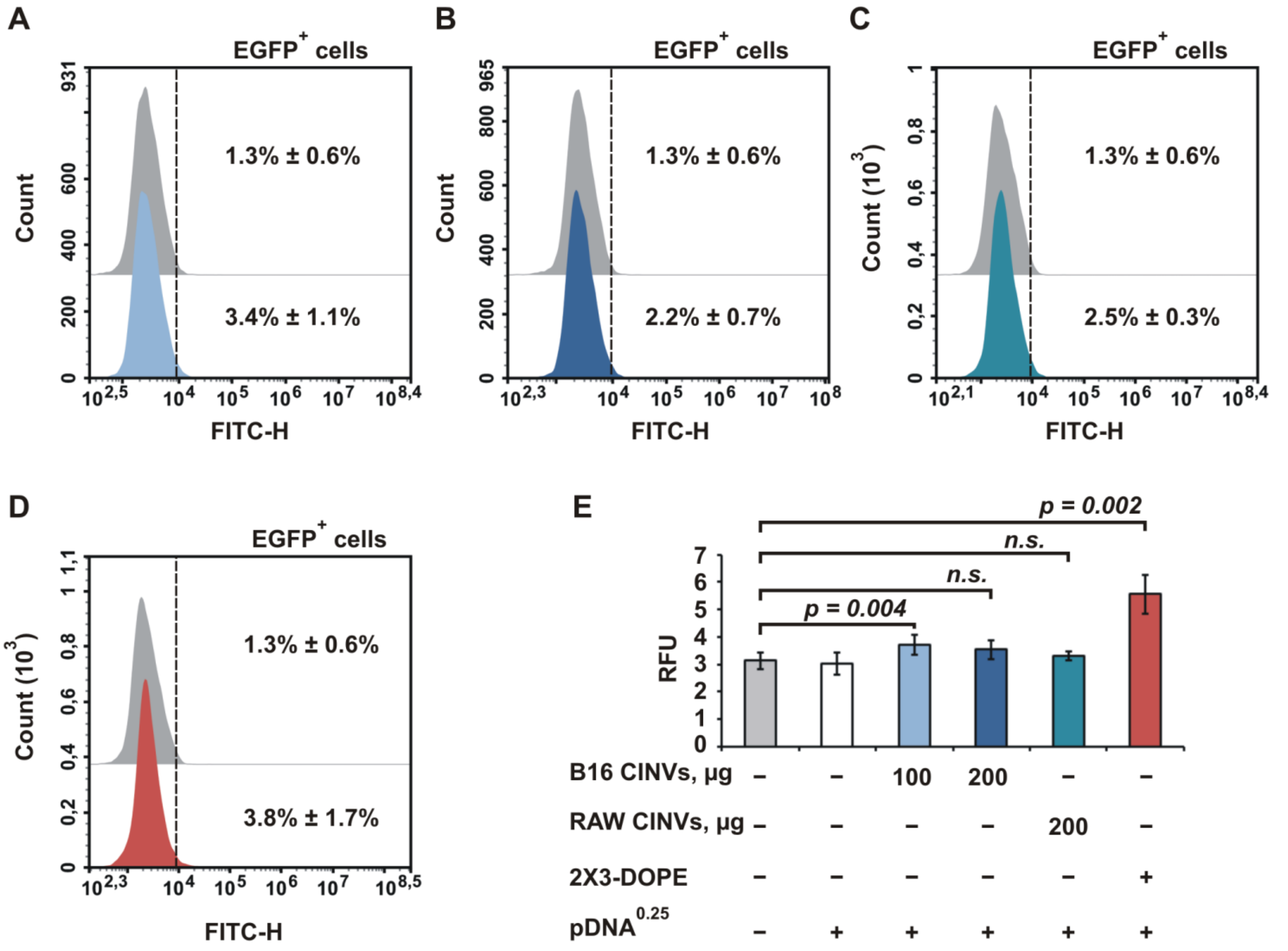

Supplementary Figure S11. Delivery of pDNA into B16 cells. Flow cytometry assay data (n = 3-11). Delivery of pDNA was performed by B16 CINVs (100 µg/well) **(A)**, B16 CINVs (200 µg/well) **(B)**, RAW CINVs (200 µg/well) **(C)**, or 2X3-DOPE liposomes **(D)**. **(E)** Summary RFU data. The superscript indicates the amount of pDNA (µg) used in the loading mixture. Data are presented as mean and SD; *n.s.*– not significant.
